# Multi-omics analysis identifies oxidative stress-related biomarkers and therapeutic targets linking periodontitis and ulcerative colitis via the oral-gut axis

**DOI:** 10.3389/fimmu.2026.1756687

**Published:** 2026-04-10

**Authors:** Lingxu Wang, Qiule Gu, Xu Ding, Chunbo Tang, Jin Wu

**Affiliations:** 1 Department of Oral Implantology, The Affiliated Stomatological Hospital of Nanjing Medical University, Nanjing, Jiangsu, China; 2 State Key Laboratory Cultivation Base of Research, Prevention and Treatment for Oral Diseases, Nanjing Medical University, Nanjing, Jiangsu, China; 3 Department of Oral and Maxillofacial Surgery, The Affiliated Stomatological Hospital of Nanjing Medical University, Nanjing, Jiangsu, China

**Keywords:** bioinformatics, machine learning, oral-gut axis, oxidative stress, periodontitis, ulcerative colitis

## Abstract

Periodontitis (PD) and ulcerative colitis (UC) are two common chronic inflammatory diseases increasingly connected via the “oral-gut axis,” yet their shared molecular mechanisms remain unclear. Oxidative stress, driven by excessive reactive oxygen species (ROS), represents a key pathogenic mechanism common to both PD and UC. In this study, we integrated transcriptomic datasets from patients with PD and UC to identify oxidative stress-related genes underlying their comorbidity. By combining weighted gene co-expression network analysis (WGCNA), machine learning, and single-cell RNA sequencing, we identified and validated a set of comorbidity-associated diagnostic biomarkers: CXCL1, XBP1, CD93, FYN, SELP, and CXCR4. These genes demonstrated high diagnostic accuracy across independent datasets, and gene set enrichment analysis (GSEA) revealed their involvement in inflammatory and immune-related pathways. Single-cell analysis further demonstrated endothelial-specific co-expression of SELP and CD93, highlighting their potential roles in intercellular communication and chronic inflammation. Moreover, molecular docking identified candidate therapeutic compounds with strong binding affinities for these targets. Collectively, our findings elucidate shared oxidative stress–driven mechanisms linking PD and UC and propose novel biomarkers and therapeutic targets for these interconnected diseases.

## 1 Introduction

Periodontitis (PD) and ulcerative colitis (UC) are major chronic inflammatory diseases that contribute significantly to the global health burden. Substantial epidemiological evidence has identified periodontitis as an independent risk factor for UC, with risk further amplified in specific populations such as elderly smokers. Clinically, this association manifests as an increased prevalence and severity of periodontal disease in UC patients relative to the general population (1, 2). These observations suggest shared pathophysiological mechanisms, conceptualized in the “oral–gut axis” hypothesis, whereby oral inflammation and microbial dysbiosis disrupt gut homeostasis, and intestinal inflammation, in turn, may exacerbate periodontal damage (3–6). Host-associated microbiomes are central to this interplay, as periodontal pathogens such as *Porphyromonas gingivalis* can translocate via the oral–gut axis and gut barrier disruption facilitates their colonization. This microbial shift acts as a primary trigger, activating pro-inflammatory cascades and mucosal impairment that collectively establish a state of sustained cellular stress (7, 8). Animal studies have provided further mechanistic support for this model, demonstrating that experimental PD can aggravate intestinal inflammation through various pathways, including microbial translocation, immune cell activation and systemic inflammation (3).

Oxidative stress, a critical pathological process characterized by an imbalance between reactive oxygen species (ROS) production and clearance, drives cellular damage, tissue destruction, and sustained chronic inflammation. Accumulating evidence highlights its central role in both the onset and progression of PD and UC. In PD, oxidative stress amplifies inflammatory responses and promotes periodontal tissue breakdown through excessive ROS accumulation and compromised antioxidant defenses, often involving suppression of the NRF2 signaling pathway (9, 10). While in UC, oxidative stress similarly disrupts intestinal barrier integrity, activates pro-inflammatory cascades, and impairs redox homeostasis. Consequently, oxidative stress functions as both an initiating trigger of inflammation and a key contributor to disease persistence and progression (11). However, the molecular basis underlying PD–UC comorbidity, particularly the specific regulatory mechanisms involving oxidative stress–related signaling pathways, remains to be systematically elucidated.

**Figure 1:**
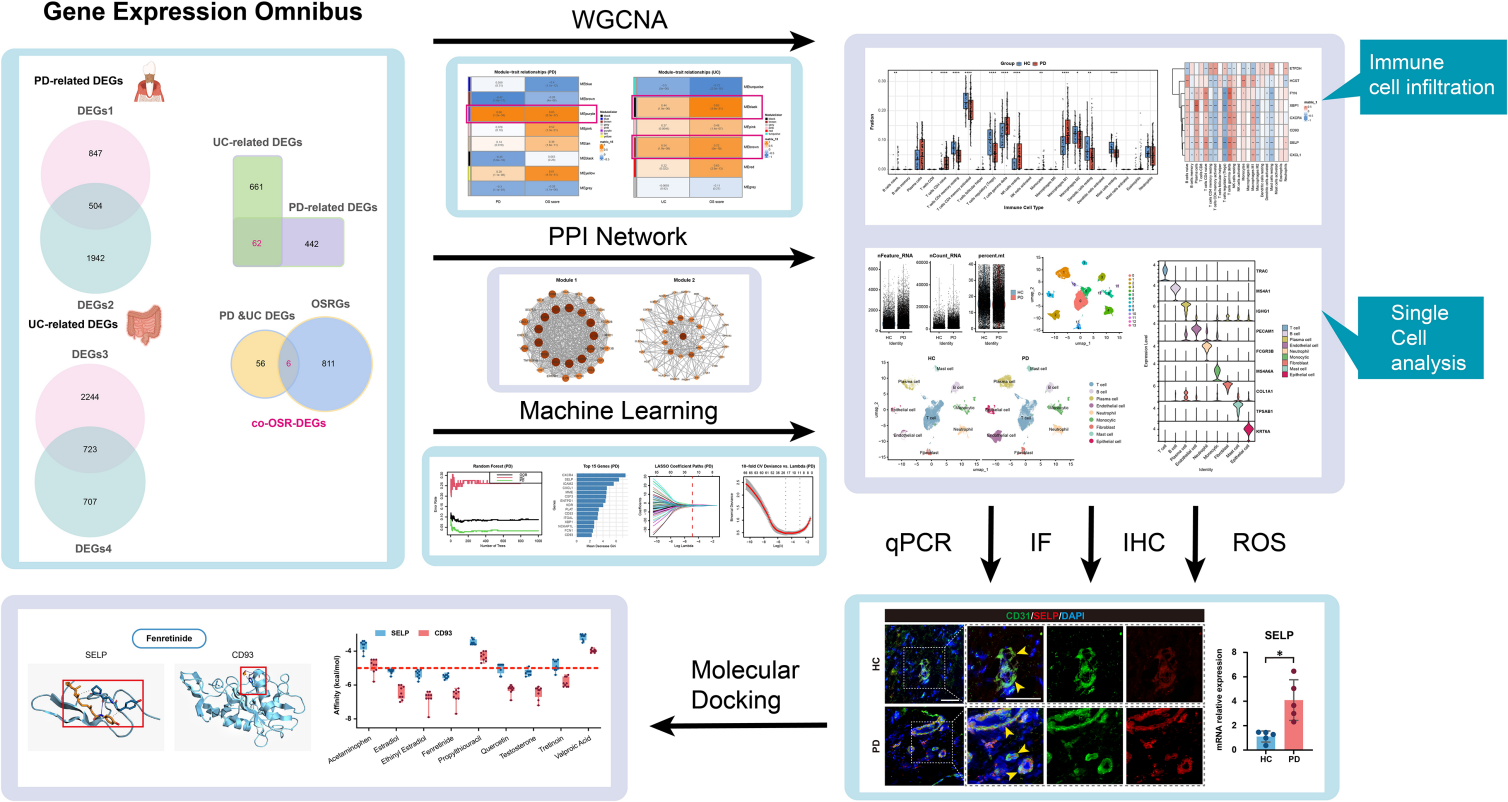
Flowchart depicting the study design.

In this study, we employed an integrative bioinformatics strategy to systematically explore the molecular mechanisms underlying the comorbidity of PD and UC. Through integrated analysis of cross-tissue transcriptomic datasets, we identified shared oxidative stress-related differentially expressed genes and further pinpointed key functional modules and hub genes using Weighted Gene Co-expression Network Analysis (WGCNA). Machine learning algorithms, including Random Forest (RF) and Least Absolute Shrinkage and Selection Operator (LASSO) regression, were then utilized to screen for robust diagnostic biomarkers. Cellular-level validation was performed via single-cell RNA sequencing, and molecular docking was employed to predict therapeutic compounds targeting the identified key proteins. This study aims to provide new insights into the comorbidity mechanism of PD and UC from an oxidative stress perspective, and to identify potential targets for developing common intervention strategies for both diseases. The methodological workflow of this study is summarized in **Figure 1**.

## 2 Method

### 2.1 Data collection and processing

To explore the potential molecular connections between PD and UC, we systematically collected transcriptomic datasets representing both disease contexts from the GEO database (https://www.ncbi.nlm.nih.gov/geo/), including GSE10334, GSE117993, and GSE126124. Specifically, the PD-related transcriptomic dataset GSE10334 comprised gingival tissues from 183 diseased sites and 64 healthy sites, while an internal dataset from our previous study (12) included 7 PD and 7 healthy gingival samples. For the UC cohort, intestinal tissue transcriptomes were obtained from GSE117993 (43 UC and 55 non-IBD samples) and GSE126124 (18 UC and 21 healthy samples). Together, these datasets provide a comprehensive foundation for cross-tissue comparative analyses between oral and intestinal inflammatory diseases. Detailed information of these datasets is provided in **Supplementary Table 1**.

### 2.2 Identification and visualization of differentially expressed genes

Differential expression analyses were performed on both PD and UC datasets using the limma R package (13). Differentially expressed genes (DEGs) were defined with a significance threshold of an adjusted P-value (FDR) < 0.05 using the Benjamini-Hochberg method and an absolute log2 fold change (|log2FC|) > 0.5. For multiple datasets corresponding to the same disease model, overlapping upregulated and downregulated genes were extracted to determine consistently dysregulated genes across datasets. Subsequently, to identify co-oxidative stress-related differentially expressed genes (co-OSR-DEGs), the overlapping DEGs between PD and UC were intersected with a comprehensive list of oxidative stress-related genes (OSRGs) retrieved from GeneCards. The extracting genes from the GeneCards database all have a relevance score≥ 7. To investigate the potential roles of oxidative stress-related key genes in PD and UC, we then submitted PD and UC Shared Oxidative Stress genes and previously reported oxidative stress-related comorbidity-associated genes (9, 14, 15) to the STRING database (https://www.string-db.org) (16).

### 2.3 Construction of weighted gene co-expression network analysis

WGCNA constructs co-expression networks based on gene expression patterns to systematically identify functionally related gene modules and associate them with phenotypic traits. This approach enables a more comprehensive discovery of potential key regulatory genes (17–21). Accordingly, we applied WGCNA to the PD dataset GSE16134 (241 PD and 69 control sites) and the UC-related dataset GSE59071 (97 UC and 11 healthy intestinal tissue samples) to further explore gene co-expression patterns under different disease states. Comprehensive details regarding these datasets are summarized in **Supplementary Table 1**. Initially, we calculated the median absolute deviation (MAD) for each gene and selected the top 5,000 genes with the highest variation for network construction. We then performed quality control on the raw expression matrix by removing low-expression genes and excluding missing or abnormal samples. Using Pearson correlation coefficients, we assessed gene expression similarity and selected an appropriate soft-thresholding power to construct a weighted adjacency matrix. We identified co-expression modules based on a topological overlap matrix (TOM) and dynamic tree cutting. Subsequently, we merged similar modules according to the correlation of module eigengenes (MEs). Finally, we correlated MEs with clinical phenotypes (disease status and oxidative stress scores) and combined gene significance (GS) with module membership (MM) to identify potential key modules and hub genes.

### 2.4 PPI and MCODE analysis

We constructed the protein-protein interaction (PPI) network using a medium confidence threshold (interaction score > 0.4) and removed disconnected nodes to ensure network connectivity. The results of PPI network into Cytoscape (version 3.10.3) was used for visualization. Using the MCODE plugin, we identified significant cluster modules in the network and selected the two highest-scoring modules as candidate core gene clusters for subsequent analysis.

### 2.5 Gene ontology and KEGG pathway enrichment analysis

We performed functional annotation and pathway enrichment analysis of candidate core genes using the clusterProfiler package. The analysis utilized the Kyoto Encyclopedia of Genes and Genomes (KEGG) to obtain functional pathway information and incorporated the Gene Ontology (GO) database for biological function annotation (22). The significance threshold was set at an adjusted P-value < 0.05 to identify statistically significant enrichment terms and pathways. Finally, we visualized the top 20 significantly enriched GO terms and KEGG pathways using the ggplot2 package.

### 2.6 Machine learning

To further identify oxidative stress-associated hub genes shared between PD and UC, we applied two machine learning algorithms: RF and LASSO regression (23–27). Using the random Forest package, we built an RF model containing 1000 decision trees based on gene expression data from disease and control groups. The model evaluated gene importance using the Mean Decrease Gini index. We selected the top 15 genes with the highest importance scores as candidate biomarkers and assessed model robustness via the Out-of-Bag (OOB) error, visualizing error rates across tree numbers. Subsequently, we constructed a LASSO logistic regression model using the glmnet package, with key module genes as independent variables and disease status (PD or UC) as the binary response. A 10-fold cross-validation determined the optimal regularization parameter λ (lambda.min), retaining only genes with non-zero coefficients as final potential diagnostic markers. Candidate diagnostic genes were identified by integrating comorbidity-associated genes from PD and UC derived using two machine learning approaches. Finally, the pROC package generated receiver operating characteristic (ROC) curves (28), and the area under the curve (AUC) was calculated to evaluate the diagnostic performance of these candidate genes.

### 2.7 Immune infiltration profiling analysis

To evaluate immune cell infiltration in PD and UC samples, we employed the CIBERSORT (29) deconvolution algorithm. Using the LM22 signature matrix with 1,000 permutations and without quantile normalization, we estimated the relative proportions of 22 immune cell subsets. An immune infiltration matrix from these results was constructed for subsequent intergroup difference and correlation analyses. The Wilcoxon rank-sum test was used to assessed differences in relative immune cell proportions between groups. Then we calculated Pearson correlation coefficients between expression levels of candidate key biomarkers and relative immune cell abundances. To control the false discovery rate in multiple hypothesis testing, we applied the Benjamini–Hochberg (BH) procedure for P-value adjustment. Based on these data, we visualized the correlation results to clearly demonstrate their differences.

### 2.8 Gene set enrichment analysis

Gene Set Enrichment Analysis (GSEA) was performed to identify pathways associated with the hub genes (30). Using the org.Hs.eg.db and clusterProfiler packages, we calculated Pearson correlation coefficients between the expression levels of key feature genes and the genome-wide expression matrix. We then ranked genes based on these correlation scores. Next, enrichment analysis was then conducted using the gseKEGG function against the KEGG pathway database (parameters: nPerm = 1000, pvalueCutoff = 0.05) to identify signaling pathways significantly associated with the disease status. The enrichment results were visualized using the enrichplot package.

### 2.9 Single-cell RNA sequencing data analysis

To investigate cell-type-specific transcriptional alterations associated with PD and UC, single-cell RNA sequencing (scRNA-seq) data were obtained from the GEO database (21), comprising datasets GSE171213 (gingival tissues from 4 healthy controls and 5 PD patients) and GSE231993 (intestinal tissues from 4 healthy controls and 4 UC patients). Raw data was processed into Seurat objects using the Seurat R package and applied quality control filters to ensure data reliability. For GSE171213, we retained cells with more than 200 detected genes and a mitochondrial gene ratio below 40% (31). For GSE231993, we kept cells with 200–6000 detected genes and a mitochondrial gene ratio under 25% (32). After quality control, 32,750 and 32,430 cells remained for subsequent analysis, respectively. We then normalized the data and identified highly variable genes. Principal component analysis (PCA) reduced dimensionality and integrated the datasets. Cell clustering was performed using the FindNeighbors and FindClusters functions, and we annotated cell types based on established marker genes.

### 2.10 Trajectory analysis

To further investigate the dynamic expression of key genes within specific cell subsets, we performed pseudotemporal analysis (33, 34). First, Seurat was used to identify highly variable genes and conduct initial clustering, constructing a single-cell expression matrix. The raw count matrix was then converted into a CellDataSet object for Monocle2 (35), followed by data normalization and dispersion estimation. We selected genes expressed in at least five cells as ordering genes and performed differential analysis using cluster information as grouping variables to identify key genes for pseudotime reconstruction. Using the DDRTree method, we embedded cells into a two-dimensional space and ordered them along a pseudotemporal trajectory to reconstruct cell developmental dynamics. For visualization, we generated cell distribution maps colored by cluster, state, or pseudotime, along with expression gradient plots of key genes across the trajectory. These visualizations facilitate observation of cell fate transitions and dynamic gene expression changes during the process.

### 2.11 Interactions between intercellular communication

We employed the CellChat (36) package to analyze cell-cell communication using single-cell RNA-seq data, systematically characterizing potential interactions between different cell types and their signaling pathway features. After constructing the CellChat object, we applied the human ligand-receptor interaction database (CellChatDB.human) to identify highly expressed genes and detect significant ligand-receptor pairs. Then the expression data was projected onto a PPI network to enhance the accuracy of signal prediction, and we calculated both intercellular communication probabilities and signaling pathway strengths. Furthermore, we used the computeCommunProbPathway function to integrate communication information for each pathway and quantify the number and intensity of network communications.

### 2.12 Patient recruitment and sample collection

To validate of the key genes in clinical samples, we collected gingival samples from 5 patients with PD and 5 healthy controls, as well as intestinal mucosal samples from 5 patients with UC and 5 healthy controls. The inclusion criteria for PD patients were: age between 18 and 65 years, a clinical diagnosis of PD (based on gingival bleeding, periodontal pocket depth ≥ 4 mm, and clinical attachment loss), no systemic diseases, no recent use of antibiotics or anti-inflammatory drugs, and no prior periodontal surgery. For UC patients, the inclusion criteria required: age 18–65 years, a confirmed diagnosis of UC by endoscopy and histopathology, absence of severe comorbidities (such as infection or cardiac, hepatic, or renal dysfunction), and no recent use of immunosuppressants or biologics. We selected healthy controls to match the patients in age and sex, with no history of PD or intestinal diseases. This study was approved by the Institutional Review Board (IRB) of Nanjing Medical University. Specifically, the collection of periodontal tissues was approved under Approval No. PJ2023-089-001, and the collection of intestinal tissues was approved under Approval No. 2023-SR-035. Written informed consent was obtained from all participants.

### 2.13 Reactive oxygen species detection

ROS levels were detected in frozen sections of periodontal tissue from PD patients and HC, as well as in intestinal mucosa from UC patients using the fluorescent probe 2′, 7′-dichlorodihydrofluorescein diacetate (DCFH-DA, Beyotime, China). Briefly, frozen sections (8 μm thickness) were prepared using a cryostat microtome, fixed in 4% paraformaldehyde for 15 minutes, and washed with phosphate-buffered saline (PBS, Gibco, USA). The sections were then incubated with 10 μM DCFH-DA at 37°C for 30 minutes in the dark. After incubation, the samples were rinsed three times with PBS to remove excess probe. Nuclei were counterstained with 4’,6-diamidino-2-phenylindole (DAPI, Beyotime, China). Fluorescence images were acquired using a Leica DM6 Microsystems (German), and ROS-positive regions were quantified based on fluorescence intensity using ImageJ software.

### 2.14 Quantitative real-time polymerase chain reaction

Total RNA was extracted from homogenized human gingival and intestinal tissue samples using TRIzol reagent (Invitrogen, USA). We synthesized cDNA with the Primescript RT Reagent (Vazyme, China). Quantitative real-time PCR (qRT-PCR) was performed on a QuantStudio Q7 Real-Time PCR System (Thermo Fisher Scientific, USA) using SYBR Premix Ex Taq (Vazyme). We normalized the expression levels of target genes to Gapdh as an internal control and calculated relative quantification using the 2^–ΔΔCt^ method. The primer sequences used in this study are listed in **Supplementary Table 2**.

### 2.15 Immunofluorescence and immunohistochemistry staining

For both immunofluorescence (IF) and immunohistochemistry (IHC) staining, fresh-frozen tissue sections were fixed, air-dried, and subsequently permeabilized. To mitigate endogenous peroxidase activity, sections were treated with 3% hydrogen peroxide. Non-specific binding sites were blocked with 5% BSA (Servicebio, China) for 1 hour at room temperature.

For IF, sections were incubated overnight at 4 °C with primary antibodies targeting SELP, CD93, and CD31. After thorough washing, fluorochrome-conjugated secondary antibodies were applied for 1 hour at room temperature, and nuclei were counterstained with DAPI prior to imaging on a Leica DM6 Microscope.

IHC was performed by incubating sections overnight at 4 °C with primary antibodies against CD93 and SELP. The following day, an HRP-conjugated IgG polymer was applied, and the signal was visualized using 3,3′-diaminobenzidine (DAB, MX, China). Nuclei were lightly counterstained with hematoxylin. A complete list of antibodies is provided in **Supplementary Table 3**.

### 2.16 Molecular docking

The Comparative Toxicogenomics Database (CTD; https://ctdbase.org/) is an innovative digital ecosystem that includes chemical-gene, chemical-phenotype, chemical-disease, gene-disease and chemical-exposure interactions to advance understanding about human health (37). “Periodontitis”, “Ulcerative”, “CD93”, and “SELP” were employed as keywords to screen compounds associated with the two diseases and genes in this database. Overlapping molecules were identified using Venny 2.1.0 (https://bioinfogp.cnb.csic.es/tools/venny) for subsequent molecular docking analysis (38). Three-dimensional structures of the selected small molecules were retrieved from PubChem (https://pubchem.ncbi.nlm.nih.gov), and target protein structures were obtained from the Protein Data Bank (PDB; https://www.rcsb.org). Water molecules and heteroions were removed, and hydrogen atoms were added to the receptors using PyMOL (v2.6). Atomic charges were then calculated with AutoDock (v1.5.7) (39). After defining the docking grid parameters based on the binding regions of the receptors and ligands, we conducted molecular docking using AutoDock Vina (v1.1.2) and computed the binding free energy (in kcal/mol).

### 2.17 Statistical analysis

All bioinformatics analyses were performed using R version 4.3.3. Statistical analyses were performed using GraphPad Prism 9. Data are presented as mean ± standard deviation (SD). Comparisons between two groups were conducted with a two-tailed unpaired Student’s t-test, while comparisons involving more than two groups were analyzed using one-way analysis of variance (ANOVA) followed by Tukey’s *post-hoc* test. A P-value < 0.05 was considered statistically significant.

## 3 Results

### 3.1 Identification of OS-related DEGs in PD and UC

**Figure 2:**
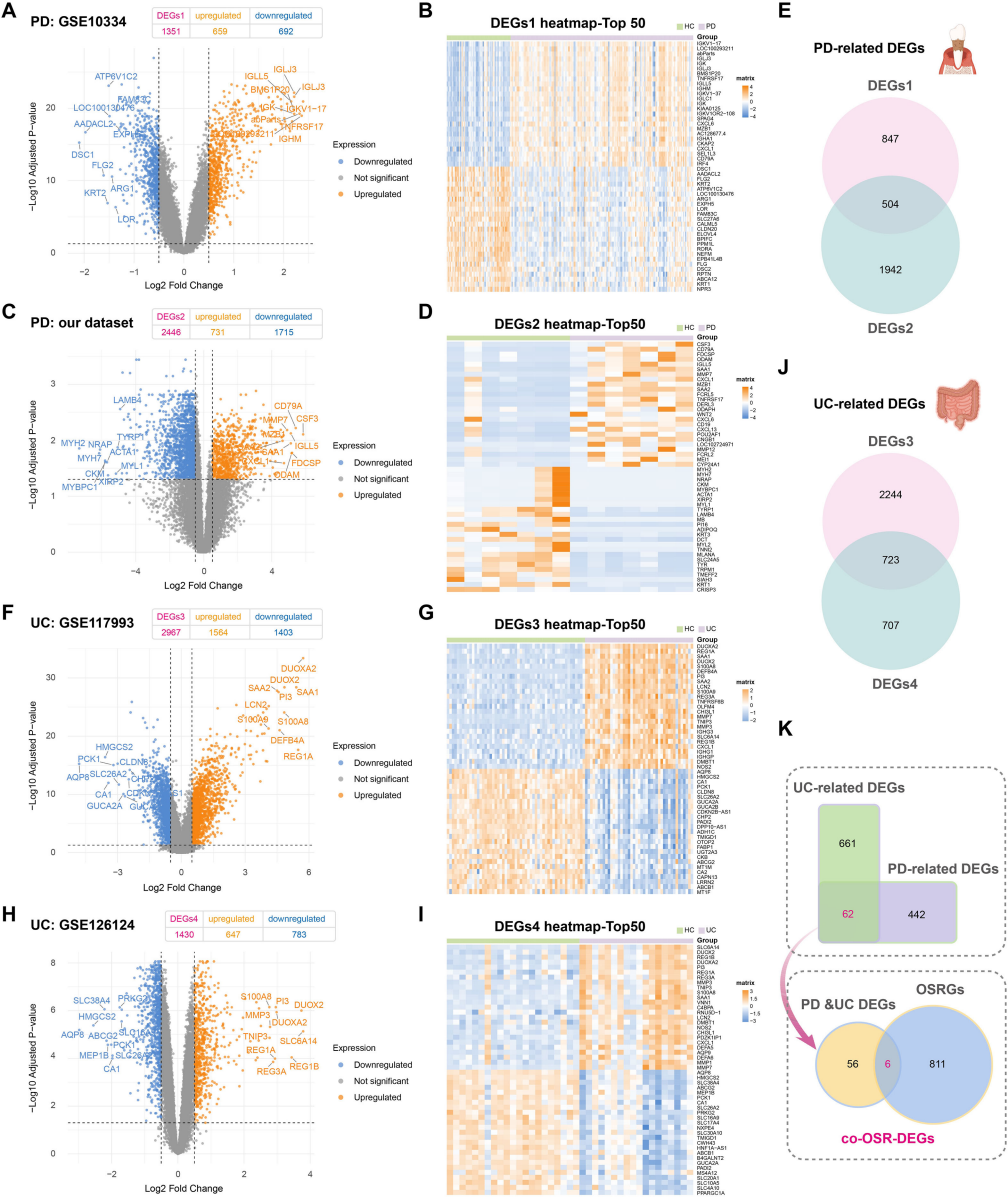
Identification of oxidative stress-related DEGs in PD and UC. **(A, B)**. Volcano plot and heatmap showing the distribution of DEGs in PD-GSE10334. Orange: upregulated; blue: down-regulated. **(C, D)**. Volcano plot and heatmap of DEGs in PD cohort from our dataset. **(E)**. Venn diagram depicting overlapping DEGs in PD. **(F, G)**. Volcano plot and heatmap of DEGs in UC-GSE117993. **(H, I)** Volcano plot and heatmap of DEGs in UC-GSE126124. J. Venn diagram depicting overlapping DEGs in UC. K. Venn diagram showing overlapping DEGs among PD, UC, and oxidative stress-related genes (OSRGs).

We performed a comparative transcriptomic analysis across multiple datasets to explore shared molecular mechanisms underlying PD and UC. From the PD datasets GSE10334 (**Figures 2A, B**) and our own datasets (**Figures 2C, D**), we identified 504 significantly dysregulated genes (**Figure 2E**). In the UC datasets GSE117993 (**Figures 2F, G**) and GSE126124 (**Figures 2H, I**), 723 differentially expressed genes were detected (**Figure 2J**). All genes were filtered using consistent thresholds (|log2FC| > 0.5 and adjusted P-value < 0.05). Further analysis revealed 62 genes that were commonly dysregulated in both PD and UC (**Figure 2K**), suggesting their potential involvement in shared pathogenic pathways.

To further investigate whether these shared transcriptional changes reflect a common oxidative stress signature, we constructed a high-confidence oxidative stress reference gene set by extracting genes with a relevance score ≥ 7 from the GeneCards database. Intersection of this curated gene set with the 62 shared DEGs yielded six high-priority candidate genes implicated in oxidative stress pathways. These candidates exhibited consistent expression trends across both disease models (**Supplementary Figures 1A–D**), reinforcing their potential role in PD-UC comorbidity. Together, by integrating cross-disease transcriptomics with a high-confidence oxidative stress gene set, we highlight six oxidative stress-related genes that may play a central role in the shared pathophysiology of PD and UC.

### 3.2 Weighted gene co-expression network analysis of PD and UC

**Figure 3:**
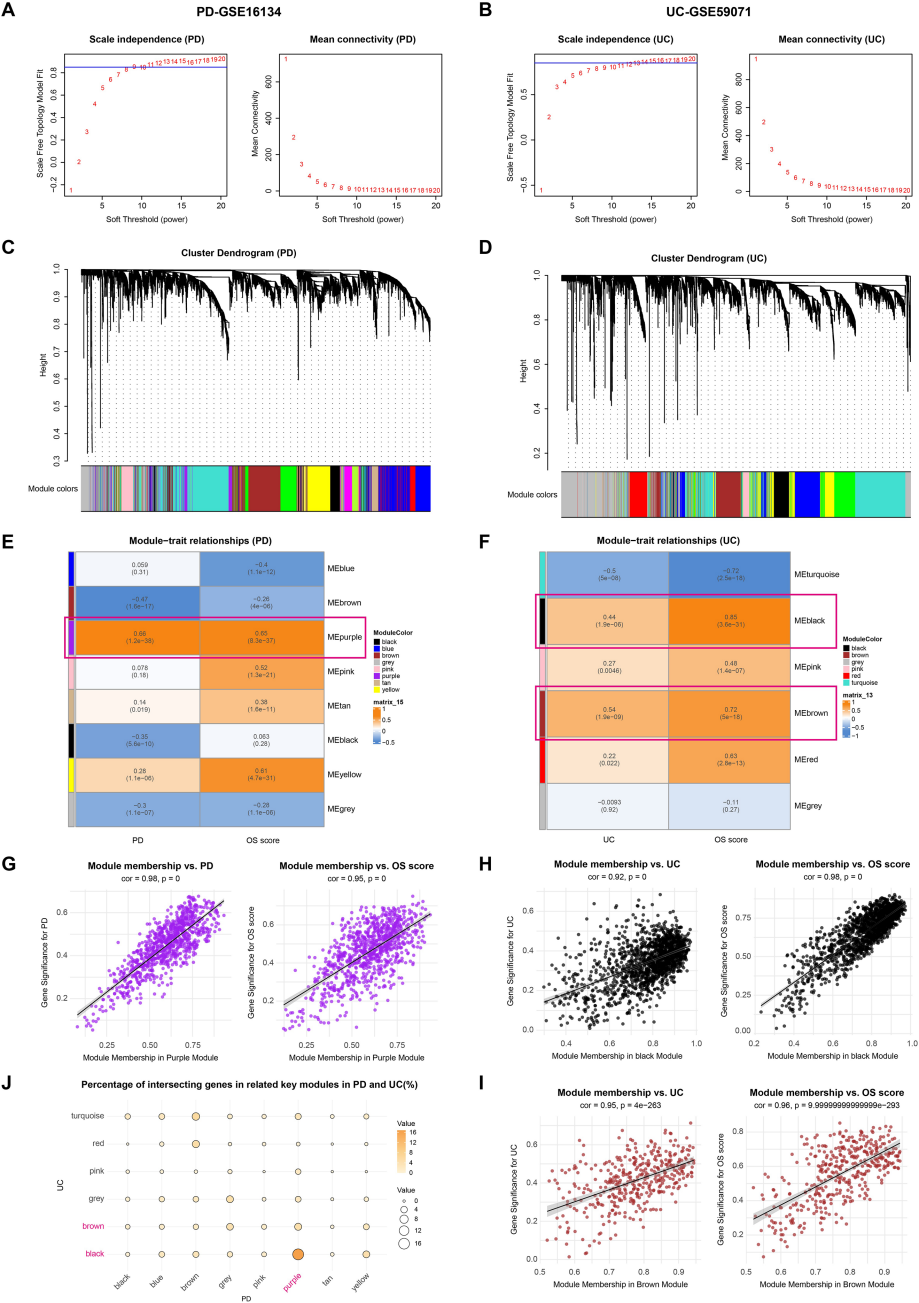
Weighted gene co-expression network analysis of PD and UC. **(A, B)**. Determination of the optimal soft-thresholding power for constructing scale-free networks in PD and UC datasets. **(C, D)**. Identification of gene modules in PD and UC datasets based on hierarchical clustering of gene expression profiles. **(E, F)**. Module–trait relationship heatmaps showing correlations between gene modules and disease phenotypes (PD or UC) as well as OS score. **(G–I)**. Scatterplots of MM versus disease phenotypes and OS score for the PD-MEpurple, UC-MEblack and UC-MEbrown modules, indicating that genes with high MM, representing core module genes, are strongly associated with the corresponding traits. **(J)**. Dot plot showing the number of overlapping genes between key modules in PD and UC, highlighting that PD-MEpurple and UC-MEblack share the largest number of common genes.

To uncover additional oxidative stress–associated genes and coordinated regulatory patterns not captured by differential expression analysis, we applied WGCNA. This method constructs a network based on pairwise correlations between genes and identifies modules of co-expressed genes, which often correspond to functional pathways or cell-type-specific signatures. This analysis was conducted on two independent datasets, GSE16134 (PD) and GSE59071 (UC), to explore associations between gene networks and clinical phenotypes (including PD and UC), as well as OS scores. After correcting for batch effects (**Supplementary Figures 2A–D**), cluster analysis grouped samples according to their intrinsic gene expression patterns, with each cluster representing a distinct set of samples. Optimal soft-thresholding powers were determined as 9 and 13 for the two datasets, resulting in 13 and 9 gene modules, respectively (**Figures 3A, B**). And then we identified gene modules in PD and UC datasets based on hierarchical clustering of gene expression profiles.

To identify gene co-expression modules associated with disease status and oxidative stress, we first calculated OS scores for each sample using ssGSEA. Gene significance scores for each module were then computed by correlating module genes with OS scores and phenotypic traits. In PD, the MEpurple module showed significant positive correlations with both disease phenotype and OS levels (**Figures 3E, G**); while in UC, the MEblack and MEbrown modules were significantly associated with disease traits and OS scores (**Figures 3F, H, I**). Notably, the MEpurple (PD) and MEblack (UC) modules shared the largest number of genes, totaling 433 (**Figure 3J**), highlighting their potential involvement in disease comorbidity. These results suggest that core genes within these co-expression networks may play key regulatory roles in disease pathogenesis. Overall, the WGCNA analysis identified phenotype- and OS-related co-expression modules across PD and UC, providing candidate gene sets for further mechanistic and therapeutic investigation.

### 3.3 PPI network construction and enrichment analysis

**Figure 4:**
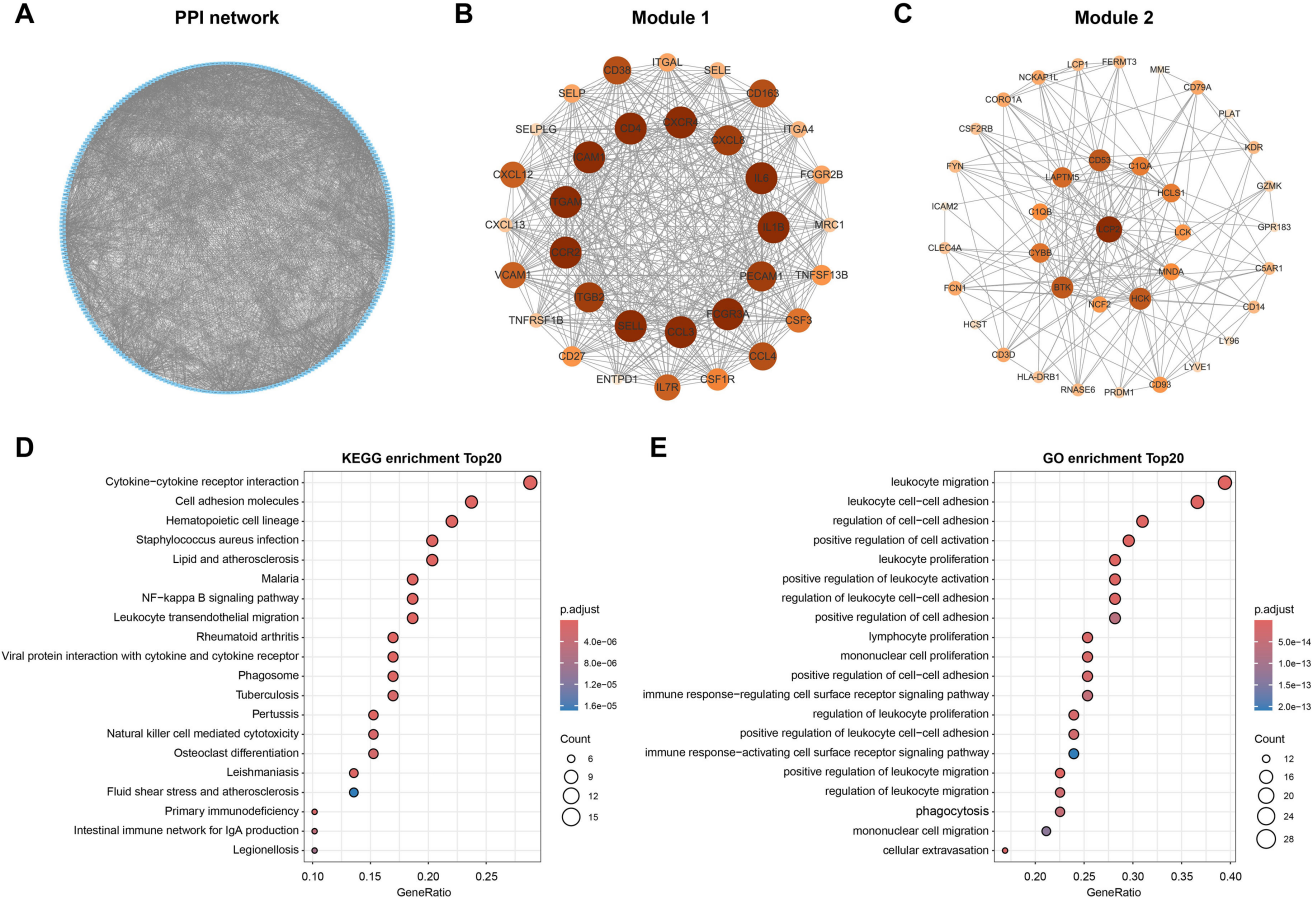
PPI network construction and enrichment analysis. **(A)**. STRING-based PPI network of the identified feature genes. **(B, C)**. PPI networks of genes from the top two high-scoring modules identified by MCODE analysis in Cytoscape. Each node represents a gene, and node size indicates its degree of centrality within the network. **(D, E)**. Dot plots illustrating the top 20 significantly enriched pathways in KEGG and GO analyses.

The epidemiological link between PD and UC suggests shared pathophysiological mechanisms, potentially involving chronic inflammation, immune dysregulation, and mucosal barrier injury (8, 40). To explore functional interactions among co-expressed genes identified in the aforementioned analyses, we constructed a PPI network using the STRING database. The analysis integrated 439 overlapping genes from the PD MEpurple and UC MEblack modules with 6 previously identified OS-related comorbidity genes. The confidence score threshold was set to 0.4 (**Figure 4A**). The resulting PPI network was imported into Cytoscape (version 3.9.1) for topological analysis and module identification. We identified 10 highly interconnected protein modules using the MCODE clustering algorithm. Based on their strong connectivity and functional coherence, we selected the two most significant modules as candidate core gene sets for functional enrichment analysis: Module 1 with 34 genes and Module 2 containing 36 genes, totaling 70 genes (**Figures 4B, C**). Subsequent KEGG and GO analyses revealed these genes to be significantly enriched in key inflammatory and immune pathways, particularly cytokine receptor interaction and NF-κB signaling (**Figures 4D, E**). These pathways are closely associated with ROS production, and their activation, together with processes such as leukocyte migration and immune cell activation, may contribute to ROS generation and regulation, establishing a feed-forward loop that exacerbates tissue damage and sustains inflammatory responses.

### 3.4 Machine learning screening of potential shared hub genes

To identify robust diagnostic biomarkers for both PD and UC, we employed two machine learning algorithms, RF and LASSO, to further refine hub genes from the candidate pools of Module 1 and Module 2 derived from the PPI network analysis.

**Figure 5:**
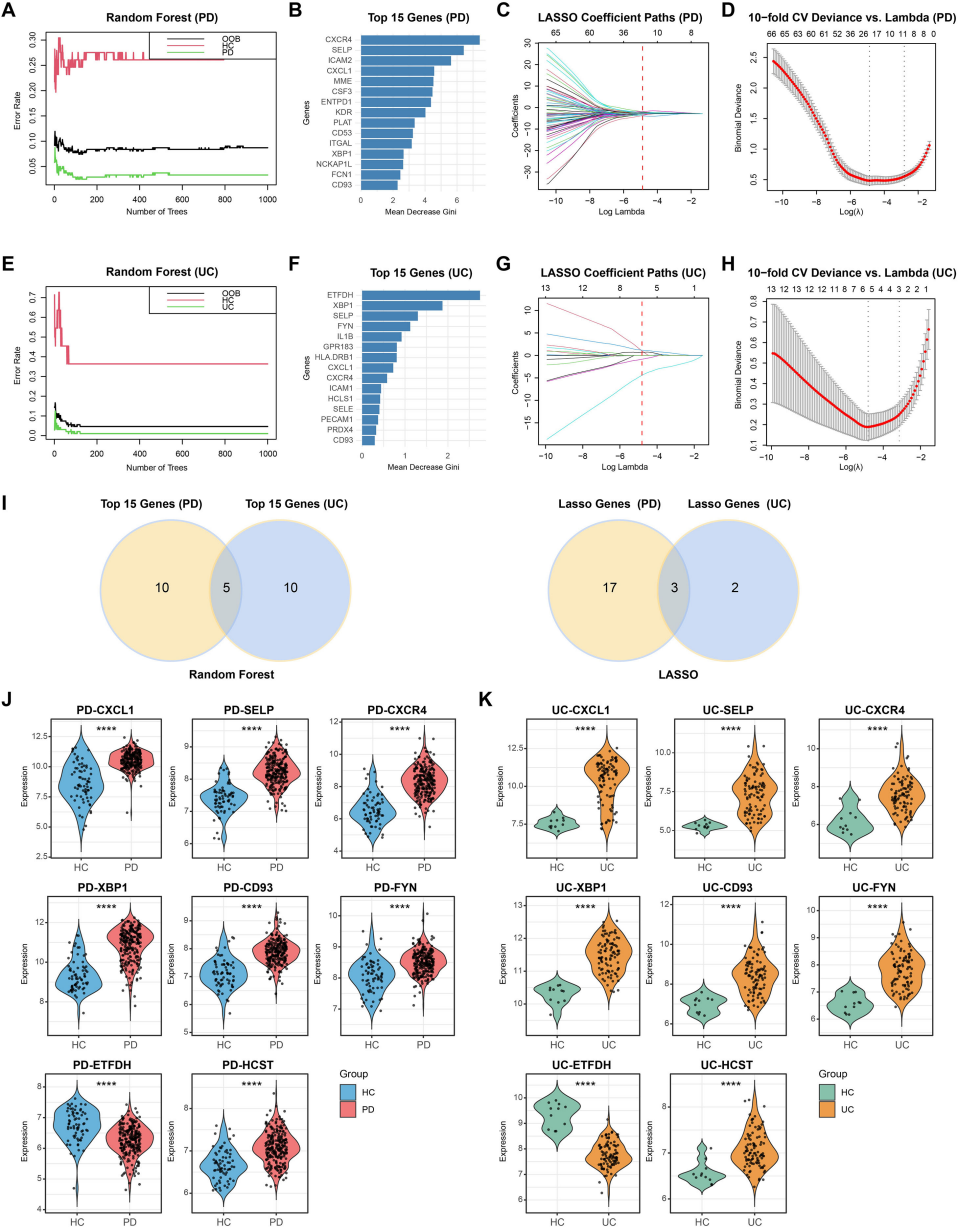
Random forest and LASSO screening for hub genes. **(A, B)**. Mean decrease in Gini index of 70 candidate genes in the PD dataset using the RF algorithm. The top 15 genes contributing most to PD classification are highlighted. **(C, D)**. Identification of the minimum binomial deviance and selection of the optimal λ value for diagnostic biomarker selection using LASSO logistic regression in the PD dataset. **(E–H)**. Application of RF and LASSO analyses to the UC dataset. **(I)**. Identification of overlapping hub genes between PD and UC based on the results of both algorithms. **(J, K)**. Expression validation of the final candidate comorbidity genes in PD and UC datasets. ****P < 0.0001.

In the PD dataset, we constructed an RF model using 70 candidate genes. As the number of trees increased, OOB error gradually stabilized, and the top 15 genes with the highest mean decrease in Gini index were selected as key pathogenic candidates (**Figures 5A, B**). Simultaneously, LASSO regression with ten-fold cross validation determined the optimal penalty parameter (λ) and identified genes with non-zero coefficients (**Figures 5C, D**). The same analytical workflow was applied to the UC dataset (**Figures 5E–H**).

Subsequently, the gene sets identified by both algorithms for PD and UC were integrated. The RF model identified five potential comorbidity-related genes, while LASSO regression selected three genes (**Figure 5I**). To enhance candidate coverage and improve identification robustness, the results from both methods were combined, yielding a final set of eight candidate genes: CXCL1, XBP1, CD93, FYN, SELP, CXCR4, ETFDH, and HCST. Expression validation confirmed these hub genes were significantly dysregulated in both PD and UC compared with healthy controls (**Figures 5J, K**), implying their potential involvement in the shared pathogenic mechanisms underlying the two diseases.

To further evaluate the diagnostic potential of these hub genes, ROC curve analysis was performed for each gene in both disease contexts. All sixteen ROC curves demonstrated high discriminatory power, with AUC values exceeding 0.70 (**Supplementary Figures 3A–P**), indicating robust diagnostic efficacy and underscoring their potential as biomarkers for PD and UC.

### 3.5 Immune cell infiltration and correlation with shared hub genes

**Figure 6:**
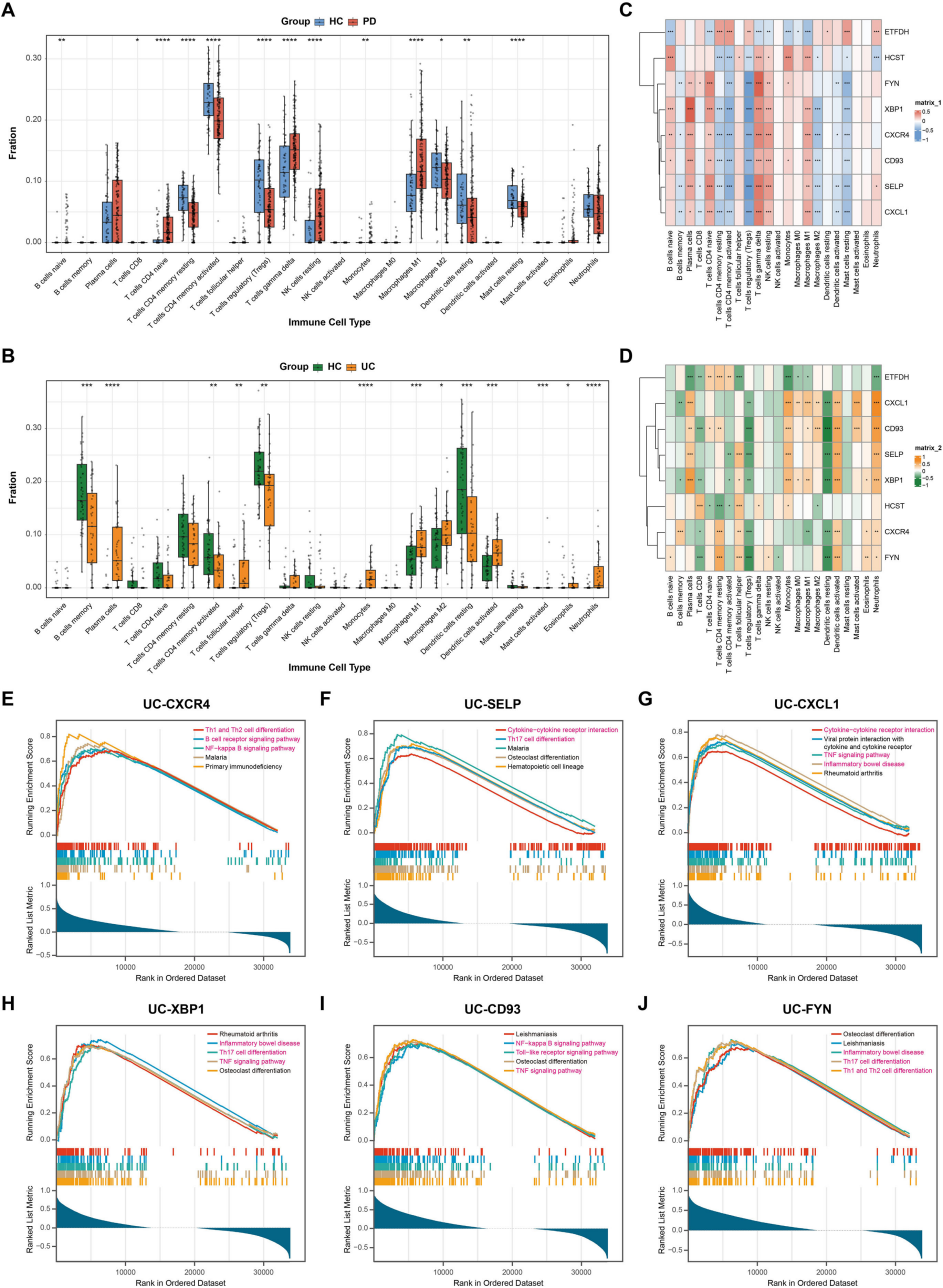
Immune cell infiltration and correlation with shared hub genes. **(A, B)**. Comparison of immune cell proportions in PD (GSE10334) and UC (GSE117993) samples estimated using CIBERSORT. **(C, D)**. Pearson correlation analysis between the shared hub genes and immune cell subsets in PD and UC. **(E–J)**. GSEA results for the hub genes in UC dataset, showing the significantly enriched biological pathways associated with inflammatory processes. *P<0.05, **P<0.01, ***P<0.001, ****P<0.0001.

PD and UC are immune-mediated diseases characterized by chronic inflammation. Given the enrichment of immune-related pathways observed in our analysis, we next investigated whether PD and UC exhibit similar immune infiltration characteristics. We employed CIBERSORT to analyze the composition of 22 immune cell subsets in PD (dataset GSE10334) and UC (dataset GSE117993) datasets. The analysis revealed an increased proportion of M1 macrophages but a decrease in M2 macrophages in PD, indicating a shift in macrophage polarization towards a pro-inflammatory phenotype. A reduction in regulatory T cells (Tregs) may weaken immunosuppression, thereby sustaining the chronic inflammatory state (**Figure 6A**). In UC, we observed a significant increase in M1 macrophages and activated dendritic cells, suggesting enhanced antigen-presenting activity. The rise in follicular helper T cells (Tfh) indicates activation of adaptive immunity, which may drive antigen-mediated inflammatory responses. Concurrently, a significant enrichment of plasma cells reflects active humoral immunity (**Figure 6B**). Overall, the immune cell infiltration patterns in PD and UC appear to share key characteristics, including sustained activation of adaptive immunity, a shift of innate immunity toward pro-inflammatory states, and a reduction in immunosuppressive function. This shared immune imbalance may be a key driver of chronic inflammation and the common pathological mechanism in both diseases.

Although differences in immune cell proportions provide initial clues to the shared pathology of PD and UC, the direct association between the shared hub genes and peripheral immune infiltration requires further validation. We therefore performed Pearson correlation analysis to evaluate the relationships between eight shared hub genes and immune cells in PD. The results showed that CXCR4, SELP, CXCL1, XBP1, CD93, FYN, and HCST correlated positively with multiple cell types, including M1 macrophages, activated CD4^+^ T cells, and plasma cells. Conversely, these genes showed significant negative correlations with M2 macrophages and Tregs (**Figure 6C**). Similarly, in UC, most shared hub genes (except ETFDH) correlated positively with neutrophils and Tfh cells, while correlating negatively with Tregs (**Figure 6D**), further underscoring the central role of immune cells in UC pathogenesis.

In order to gain deeper insight into the potential functional roles of these hub genes in signaling pathways, we performed GSEA and visualized the top five enriched pathways. Notably, in both PD and UC datasets, high-expression groups of CXCR4, SELP, CXCL1, XBP1, CD93, and FYN were predominantly enriched in inflammation-related pathways, such as Th1 and Th2 cell differentiation, B cell receptor signaling pathway, NF-κB signaling pathway, Cytokine-cytokine receptor interaction, and Th17 cell differentiation. In contrast, ETFDH associated with ribosomal biogenesis and protein synthesis pathways, such as Ribosome biogenesis and Aminoacyl-tRNA biosynthesis; while HCST involved in multiple regulatory processes, including signal transduction (Inositol phosphate metabolism), epigenetic regulation (Polycomb repressive complex), tissue organization (Hematopoietic cell lineage), and immune function (Natural killer cell mediated cytotoxicity) (**Figures 6E–J**, **Supplementary Figures 4A–J**).

Based on these analyses, we finally selected six hub genes for subsequent investigation, including CXCR4, SELP, CXCL1, XBP1, CD93, and FYN. Collectively, these shared hub genes show significant enrichment in inflammation-related pathways like cytokine signaling and T cell differentiation, while each also maintains distinct functional properties, participating variously in immune activation, signal transduction, and tissue remodeling.

### 3.6 Single-cell analysis of cellular localization and signaling roles of shared hub genes

**Figure 7:**
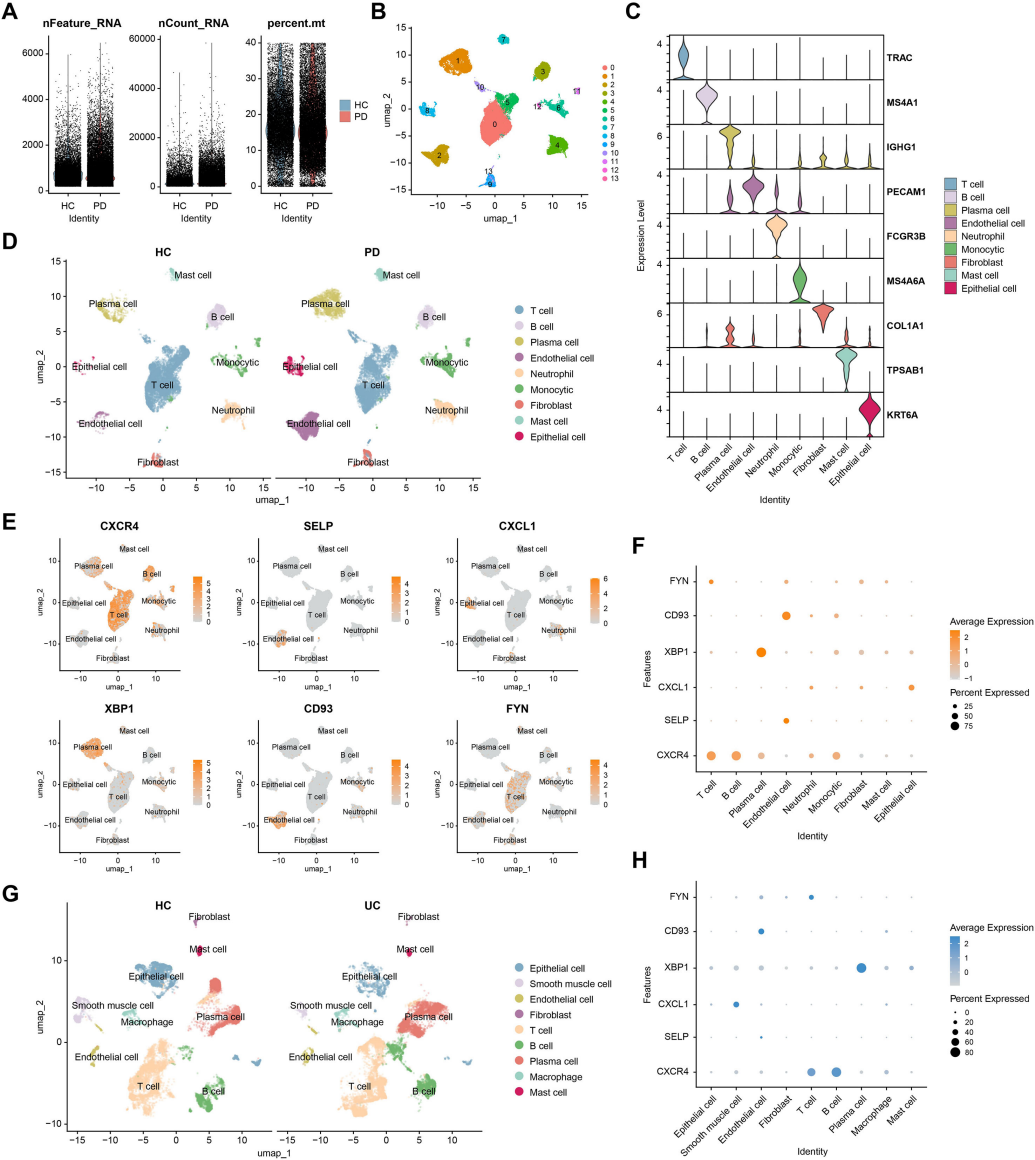
Single-cell RNA-seq analysis validating hub gene expression and cellular localization. **(A)** Distribution of nFeature_RNA, nCount_RNA, and percent.mt in gingival samples from HC and PD patients. **(B)** UMAP visualization showing the classification of cells into 14 distinct clusters. **(C)** Violin plots of canonical marker genes for cell type annotation. **(D)** UMAP visualization comparing cell type composition between HC and PD samples. **(E)** Expression patterns of CXCR4, SELP, CXCL1, XBP1, CD93, and FYN across different cell types. **(F)** Dot plot showing predominant expression of SELP and CD93 in endothelial cells within the PD microenvironment. **(G)** UMAP visualization comparing cell type composition between HC and UC samples. **(H)** Predominant expression of SELP and CD93 in endothelial cells within UC microenvironment.

To investigate the cellular basis of shared inflammatory mechanisms in PD and UC and to capture cell type-specific expression patterns, we examined two independent single-cell RNA-seq datasets, GSE171213 (PD) and GSE231993 (UC). In the PD dataset, dimensionality reduction, clustering, and normalization identified 14 cell subsets (**Figures 7A, B**). Using classical marker genes, we annotated nine major cell types: T cells, B cells, plasma cells, endothelial cells, neutrophils, monocytes, fibroblasts, mast cells, and epithelial cells (**Figures 7C, D**). UMAP-based gene expression analysis revealed distinct distribution patterns of CXCR4, SELP, CXCL1, XBP1, CD93, and FYN across cell types: CXCR4 was highly expressed in T and B cells; SELP and CD93 localized mainly to endothelial cells; CXCL1 predominated in epithelial cells; XBP1 was elevated in plasma cells; and FYN was detected in T cells (**Figure 7E**). A bubble plot further confirmed marked co-expression of SELP and CD93 in endothelial cells (**Figure 7F**), suggesting their potential cooperative role in endothelial regulation. Analysis of the UC dataset using the same single-cell workflow identified nine major cell clusters (**Supplementary Figures 5A–C**; **Figure 7G**). Both UMAP and bubble plots again revealed strong co-expression of SELP and CD93 in endothelial cells (**Supplementary Figure 5D**; **Figure 7H**), indicating this pattern is conserved across PD and UC and may reflect a shared pathogenic mechanism.

**Figure 8:**
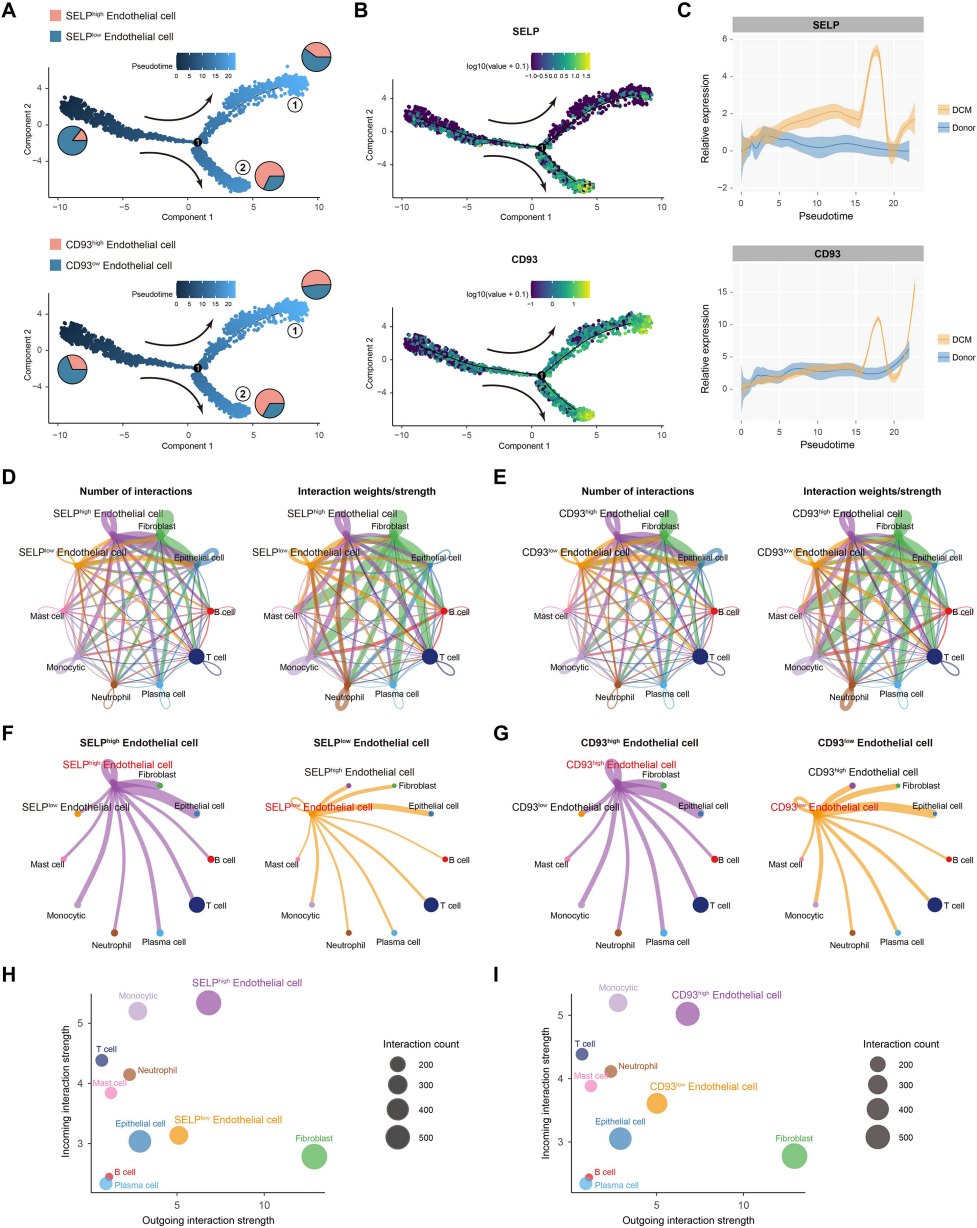
Cell trajectory and intercellular communication analysis of SELP^high^ and CD93^high^ endothelial cells in PD lesions. **(A, B)**. Quasitemporal trajectory analysis revealed the expression patterns of SELP and CD93 across endothelial cells, suggesting a coordinated progression along the differentiation path. **(C)**. Two-dimensional pseudotime plots depicting dynamic changes in SELP and CD93 expression during PD progression. **(D–G)**. Circle plots showing the numbers and strengths of intercellular communication events among SELP^high^ endothelial cells, SELP^low^ endothelial cells, CD93^high^ endothelial cells, CD93^low^ endothelial cells, and other cell types within the PD microenvironment. Colors denote interacting cell types, and edge width represents relative interaction strength. **(H, I)**. Scatter plots depicting the outgoing and incoming interaction strengths of SELP^high^ endothelial cells, SELP^low^ endothelial cells, CD93^high^ endothelial cells, and CD93^low^ endothelial cell subsets.

Vascular endothelial cells play central roles in inflammatory responses and barrier regulation, and their dysfunction underlies multiple chronic inflammatory diseases (41). While they are not the most numerically dominant cell type, endothelial cells are uniquely positioned to modulate leukocyte recruitment and the systemic translocation of inflammatory mediators, processes central to the oral–gut axis. To explore the potential involvement of shared hub genes in endothelial pathology, we examined the dynamic expression of SELP and CD93 during endothelial differentiation in PD using pseudotime trajectory analysis. Both genes exhibited coordinated upregulation, peaking at a common terminal stage (**Figures 8A–C**), which may represent a pathologically activated endothelial state with pro-inflammatory and dysregulated angiogenic features. Specifically, high SELP expression may facilitate leukocyte adhesion and recruitment during inflammation (42). Notably, CD93 is implicated in endothelial cell migration, angiogenesis, and maintenance of vascular integrity (43). However, under pathological conditions, the CD93-high subpopulation may contribute to impaired neovascular barrier function, highlighting a potential mechanism underlying vascular dysfunction in chronic inflammatory diseases (44).

Furthermore, cell–cell communication analysis based on ligand–receptor pairs revealed that SELP-high and CD93-high endothelial cells in PD significantly engaged in bidirectional signaling with multiple immune cell types, exhibiting increased interaction numbers and intensity (**Figures 8D–I**). SELP and CD93 acted as central nodes in these networks, indicating they may help amplify and sustain inflammatory signals by modulating endothelial-immune crosstalk.

In conclusion, our single-cell analyses identify characteristic endothelial overexpression of SELP and CD93 in both PD and UC, and highlight their involvement in enhanced intercellular communication networks associated with disease-related immune responses. These findings provide new cellular evidence for exploring endothelial dysfunction as a shared mechanism in these two chronic inflammatory conditions.

### 3.7 Validation of the key genes in clinical samples

**Figure 9:**
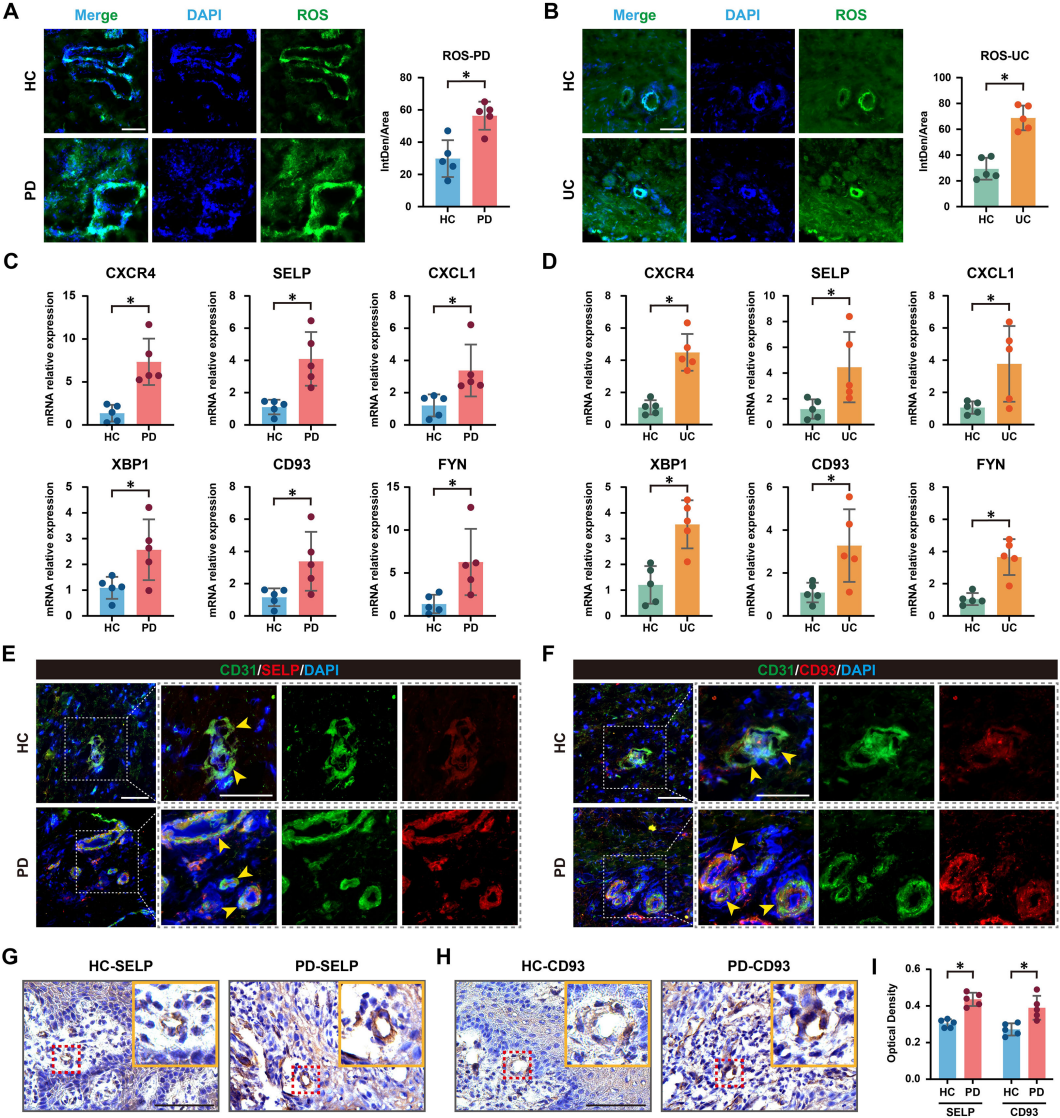
Validation of oxidative stress and the expression of SELP and CD93 in clinical samples. **(A)** Representative fluorescence images and quantitative analysis of ROS levels in gingival tissues from PD patients and healthy controls. **(B)** ROS levels in intestinal tissues from UC patients and healthy controls. **(C, D)**. qRT-PCR analysis of hub gene expression in gingival tissues from PD patients and intestinal tissues from UC patients, compared with healthy controls. **(E, F)**. IF staining showing colocalization of SELP/CD93 (red) with the endothelial marker CD31 (green) in PD gingival lesions. Nuclei were counterstained with DAPI (blue). **(G–I)**. IHC staining and quantitative assessment of SELP and CD93 expression in healthy and PD samples. Scale bar = 50 μm. Data are presented as mean ± SD (n = 5); *P < 0.05.

To demonstrate the potential link between oxidative stress and endothelial activation, we assessed ROS levels in lesional tissues from PD and UC patients using fluorescence staining. Both PD gingival (**Figure 9A**) and UC intestinal tissues (**Figure 9B**) exhibited elevated ROS levels compared to healthy controls. Interestingly, ROS signals were enriched in perivascular regions, suggesting a spatial association with endothelial regions. These findings provide morphological evidence that oxidative stress may contribute to microvascular endothelial dysfunction in inflammatory conditions.

We next validated the mRNA expression patterns of shared hub genes previously identified through bioinformatics screening, including CXCR4, SELP, CXCL1, XBP1, CD93, and FYN. qRT-PCR analysis confirmed significant upregulation of these genes in both PD (**Figure 9C**) and UC (**Figure 9D**) lesions compared with healthy tissues, supporting their involvement in disease pathogenesis.

To further characterize the protein-level expression and cellular localization of endothelial-specific targets, we performed IF and IHC assays. IF staining demonstrated clear colocalization of SELP and CD93 with the endothelial marker CD31 in both PD gingival (**Figure 9E**) and UC intestinal lesions (**Figure 9F**), confirming their predominant localization in vascular endothelial cells. Consistently, IHC results further revealed enhanced expression of SELP and CD93 specifically within the vascular endothelium of diseased tissues (**Figures 9G–I**), reinforcing their endothelial-enriched expression under pathological conditions.

Collectively, these experimental results form a coherent narrative with our preceding analyses: earlier bioinformatic findings indicated enrichment of SELP and CD93 in endothelial activation pathways shared by PD and UC, while the current multi-technique validation demonstrates their disease-specific upregulation at both transcript and protein levels, along with definitive endothelial localization. The observed perivascular ROS accumulation further suggests oxidative stress as a potential upstream regulator of endothelial activation and core gene expression, thereby laying the groundwork for future investigation into the “oxidative stress-SELP/CD93-endothelial dysfunction” axis in the comorbidity of PD and UC.

### 3.8 *In silico* validation of the targets using molecular docking

To further identify potential therapeutic strategies targeting the pro-inflammatory phenotype associated with PD and UC, we queried the CTD with “Periodontitis”, “Ulcerative”, “CD93”, and “SELP”, identifying 2549, 5995, 195, 110 compounds respectively. And the Venn diagram program revealed 47 compounds associated with CD93 and SELP among the two diseases. After screening out environmental contaminants, laboratory chemicals, and other complex mixtures from these compounds, we identified 9 compounds as the therapeutic candidates, including Quercetin, Tretinoin, Estradiol, Testosterone, Acetaminophen, Propylthiouracil, Fenretinide, Ethinyl Estradiol and Valproic Acid.

**Figure 10:**
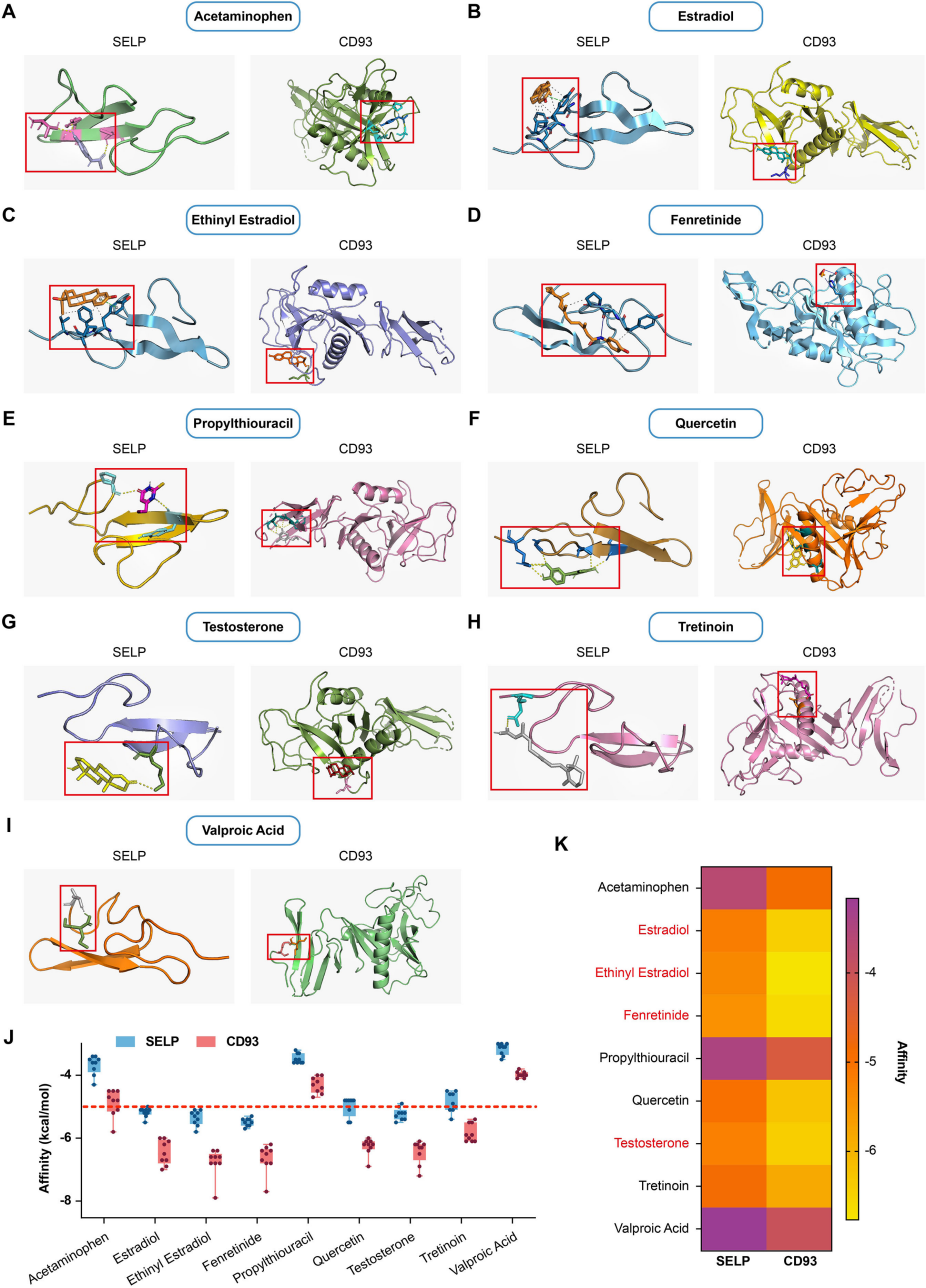
*In silico* validation of the targets using molecular docking. **(A–I)**. Two-dimensional interaction diagrams of the nine candidate small-molecule compounds docked with SELP and CD93. **(J)**. The binding affinities (kcal/mol) of the nine compounds for SELP and CD93. The dashed line represents the -5 kcal/mol threshold, with values below this threshold indicating favorable binding potential. **(K)**. Heatmap summarizing the binding energies from the molecular docking analysis. Lighter colors denote lower binding energy values, indicating stronger predicted binding affinities.

Then we conducted molecular docking analyses to assess the binding interactions between nine selected small-molecule compounds and two hub genes, SELP and CD93 (**Figures 10A–I**). Binding affinity was evaluated based on binding free energy values, where lower values (typically < -5 kcal/mol) indicate stronger binding capacity (45). All compounds except acetaminophen, propylthiouracil, and valproic acid exhibited binding affinities below -5 kcal/mol with both targets (**Figure 10J**), suggesting their potential for effective binding. The molecular docking results were visualized using PyMOL for structural analysis.

Among these candidates, Fenretinide and Ethinyl Estradiol stand out as the optimal compounds. For Fenretinide, its binding energies with SELP and CD93 are significantly below -5 kcal/mol (**Figure 10J**), and the corresponding regions in the heatmap (**Figure 10K**) are very light, indicating strong and top-tier binding potential; it binds to both SELP and CD93, the key mediators of pro-inflammatory phenotypes, making it a core candidate for regulating activated endothelial cell subtypes. Ethinyl Estradiol shows binding energies below -5 kcal/mol with both SELP and CD93 (**Figure 10J**), and the regions for both targets in the heatmap (**Figure 10K**) are relatively light, demonstrating high and tight binding affinity; it can potentially alleviate the pro-inflammatory phenotype by regulating SELP and CD93, serving as an important therapeutic candidate.

## 4 Discussion

PD is an inflammatory disease driven by dental plaque biofilm, which progressively destroys tooth-supporting tissues. UC is a chronic inflammatory disorder of the colon and rectum with variable severity among individuals. A heightened co-occurrence of PD and UC has been increasingly documented (46, 47). Periodontal pathogens have been shown to exacerbate UC under certain conditions (40). However, these findings largely rely on clinical or serological assessments, and their genetic underpinnings remain unclear (46, 48, 49). As a result, targeted therapies addressing specific genes and pathways are urgently needed. And further research is essential to reveal the distinct cellular and molecular features of these diseases.

Using a machine learning-aided bioinformatics approach, we discovered that although PD and UC are chronic inflammatory diseases of distinct anatomical locations, they share several core molecular pathways—especially in inflammatory response and oxidative stress. This supports the established hypothesis linking inflammatory bowel disease to systemic inflammation. Meanwhile, it also provides molecular evidence for the “oral-gut axis”.

We analyze the shared DEGs between PD and UC and integrate oxidative stress-related gene sets from the GEO databases. Through WGCNA, PPI network analysis and machine learning methods—including RF and LASSO regression—we selected six core genes: CXCL1, XBP1, CD93, FYN, SELP, and CXCR4. These genes are consistently dysregulated in both diseases, which indicates their involvement in a shared oxidative stress mechanism. This shared oxidative stress mechanism may contribute to tissue damage and disease progression in PD and UC. Moreover, the consistent expression pattern of these oxidative stress-related genes further suggests that oxidative stress serves as a common pathological link between the two conditions. ROC curve analysis confirmed that these genes effectively distinguish disease states (AUC > 0.70), highlighting their potential as cross-disease biomarkers. And these findings may aid in early diagnosis, patient stratification and treatment response evaluation of these two diseases.

In this study, our single-cell analysis and validation focused on vascular endothelial cells. While epithelial cells and fibroblasts are traditionally considered the primary cell types in periodontal pathophysiology due to their roles in barrier maintenance and tissue remodeling, our data-driven findings highlighted the endothelial compartment as a critical interface between PD and UC. Endothelial cells are uniquely positioned to regulate systemic leukocyte recruitment and the translocation of inflammatory mediators, making them central to the oral–gut axis and the dissemination of oral-derived signals to distant intestinal tissues. Moreover, they are preferentially targeted by oxidative stress, forming a pro-inflammatory feedforward loop central to this study (31, 50). These observations support the rationale for focusing on endothelial cells as a pivotal node.

Using single-cell transcriptomics, we identified that genes such as SELP and CD93 showed high EC-specific expression and were upregulated upon inflammatory activation. This suggests the presence of an inflammation-activated endothelial subpopulation that may sustain chronic inflammation by enhancing immune cell recruitment and vascular permeability. Cell–cell communication analysis highlighted strengthened interactions between ECs and immune cells, with SELP and CD93 as central nodes. Using the SELP–CD93 signature, we screened the Connectivity Map and identified candidate compounds to reverse this pro-inflammatory phenotype. Collectively, these findings elucidate shared mechanisms between PD and UC and suggest that ECs, as well as genes such as SELP and CD93, may represent promising therapeutic targets for chronic inflammatory diseases. Notably, the functional role of CD93 appears context-dependent. Previous studies have reported that CD93 supports tissue repair during acute inflammation by promoting angiogenesis and clearing apoptotic cells; however, chronic or sustained overexpression may impair vascular maturation and disrupt the immune microenvironment (51). Therefore, therapeutic strategies targeting CD93 should be carefully designed to preserve its beneficial functions while mitigating potential detrimental effects.

The link between PD and UC should be viewed within the broader context of the oral–gut axis, a critical conduit linking oral dysbiosis to systemic health. Recent evidence indicates that periodontal pathogens and their metabolites extend beyond localized tissue destruction, engaging in complex immune–metabolic crosstalk and translocating to the gut and systemic circulation. These processes can trigger inflammatory cascades that contribute not only to intestinal inflammation but also to systemic conditions, including metabolic and cardiovascular disorders (52, 53). In our study, the identification of endothelial dysfunction as a shared feature further reinforces this mechanistic link, suggesting that the vascular compartment may facilitate the systemic dissemination of oral-derived inflammatory signals. By situating the PD–UC connection within this oral–gut–systemic framework, our findings underscore the potential of targeting oral health as an integrative strategy to manage systemic inflammatory burden.

Taken together, in contrast to previous studies that have largely focused on individual disease mechanisms (54, 55), our study offers a transcriptome-level comparative analysis of PD and UC, aiming to identify shared pathogenic pathways beyond localized inflammatory processes. Comprehensive transcriptomic comparison highlighted overlapping inflammation and oxidative stress pathways in PD and UC. Importantly, a multi-gene panel encompassing these targets demonstrated potential for both diagnostic and therapeutic applications, offering advantages over traditional single-molecule biomarkers and providing insights into endothelial involvement in the comorbidity of PD and UC.

Nevertheless, certain limitations should be considered when interpreting the findings of this study. The transcriptomic datasets were derived from independent cohorts and lack comorbidity annotations, precluding direct investigation of patient-level co-occurrence; therefore, the shared molecular signatures identified here reflect potential biological commonalities rather than direct evidence of co-occurrence. In addition, publicly available datasets may include variations in sample processing, platform technologies, and patient demographics, which could affect reproducibility. Experimental validation in well-controlled clinical cohorts is needed to strengthen the reliability and generalizability of these signatures. Future studies should also prioritize integrated cohorts including patients with concurrent PD and UC to validate the biomarkers and further elucidate causal mechanisms along the oral–gut axis. Finally, the predicted small-molecule compounds require *in vitro* and *in vivo* assessment to confirm their efficacy and biosafety.

## 5 Conclusion

In conclusion, this study integrated an multiple bioinformatics framework, combining differential expression analysis, WGCNA, PPI network construction, and machine learning modeling, to systematically elucidate shared molecular mechanisms of inflammation and oxidative stress in PD and UC. We identified six common hub genes with high diagnostic potential, including CXCL1, XBP1, CD93, FYN, SELP, and CXCR4, which may serve as auxiliary biomarkers for both diseases. The pathways associated with these genes, including oxidative stress, cytokine signaling, and immune regulation, provide mechanistic insights into the shared pathophysiology of PD and UC. Single-cell transcriptomic analysis further highlighted endothelial cells as central mediators of immune recruitment and chronic inflammation, with SELP and CD93 representing promising therapeutic targets. Together, these findings advance our understanding of PD–UC comorbidity and offer a foundation for the development of multi-gene diagnostic tools and targeted therapeutic strategies, revealing a close molecular association and shared pathological basis between PD and UC.

## Data Availability

The raw data supporting the conclusions of this article will be made available by the authors, without undue reservation.
