# Supplementary material for: Multi-omics analysis identifies oxidative stress-related biomarkers and therapeutic targets linking periodontitis and ulcerative colitis via the oral-gut axis
Source: Front Immunol. 2026 Apr 10;17:1756687. doi: 10.3389/fimmu.2026.1756687 (PMC13105882; doi:10.3389/fimmu.2026.1756687)
Supplement: Supplementary file 1 [file DataSheet1.docx]

Supplementary Material

**Supplementary Table 1. Information on the Datasets.**

| **GSE series** | **Platform** | **Purpose** | **Tissue** | **Case (PD/UC)** | **Healthy Control (HC)** |
| --- | --- | --- | --- | --- | --- |
| GSE10334 | GPL570 | DEGs Identification | gingival tissues | 183 | 64 |
| Our dataset | - | DEGs Identification | gingival tissues | 7 | 7 |
| GSE117993 | GPL16791 | DEGs Identification | intestinal tissue | 43 | 55 |
| GSE126124 | GPL6244 | DEGs Identification | intestinal tissue | 18 | 21 |
| GSE16134 | GPL570 | WGCNA | gingival tissues | 241 | 69 |
| GSE59071 | GPL6244 | WGCNA | intestinal tissue | 97 | 11 |

**Supplementary Table 2. Primer sequences used for qRT-PCR.**

| **Gene name** | **Forward(5’-3’)** | **Reverse(5’-3’)** |
| --- | --- | --- |
| CXCR4 | GCATCTGGAGAACCAGCG | GAAACAGGGTTCCTTCATGG |
| SELP | TGGCAAGTGGAATGATGAGC | GCAGGTGTAGTTCCCGATGG |
| CXCR1 | TCTTGGCACGTCATCGTGTT | GCCAGATCACCTTCCACACA |
| XBP1 | AGCTTTTACGAGAGAAAACTCAT | ACTGGGTCCAAGTTGTCCAG |
| CD93 | GCTGTTGCTCTTATCTGCAAGGTG | AGCAAGCCTTTGCAGGGATCTA |
| FYN | ACAAAACTGACGGAGGAGAGG | GAAGCTGGGGTAGTGCTGAG |

**Supplementary Table 3. Antibodies used for IF and IHC.**

| **Category** | **Target** | **Host Species** | **Tissue** | **Application** | **No.** | **Dilution** |
| --- | --- | --- | --- | --- | --- | --- |
| Primary Antibodies | CD31 | Mouse | Proteintech | IF | 66065-2-1g | 1:500 |
|  | CD93 | Rabbit | Proteintech | IF/IHC | 18283-1-AP | 1:500 |
|  | SELP | Rabbit | Proteintech | IF/IHC | 13304-1-AP | 1:500 |
| Secondary Antibodies | FITC-anti-Mouse IgG | Goat | Abbkine | IF | A22110 | 1:200 |
|  | Cy3-anti-Rabbit IgG | Goat | Abbkine | IF | A22220 | 1:200 |
|  | HRP-conjugated anti-Mouse/Rabbit IgG (H+L) | Goat | Abbkine | IHC | A21010 | 1:200 |

**
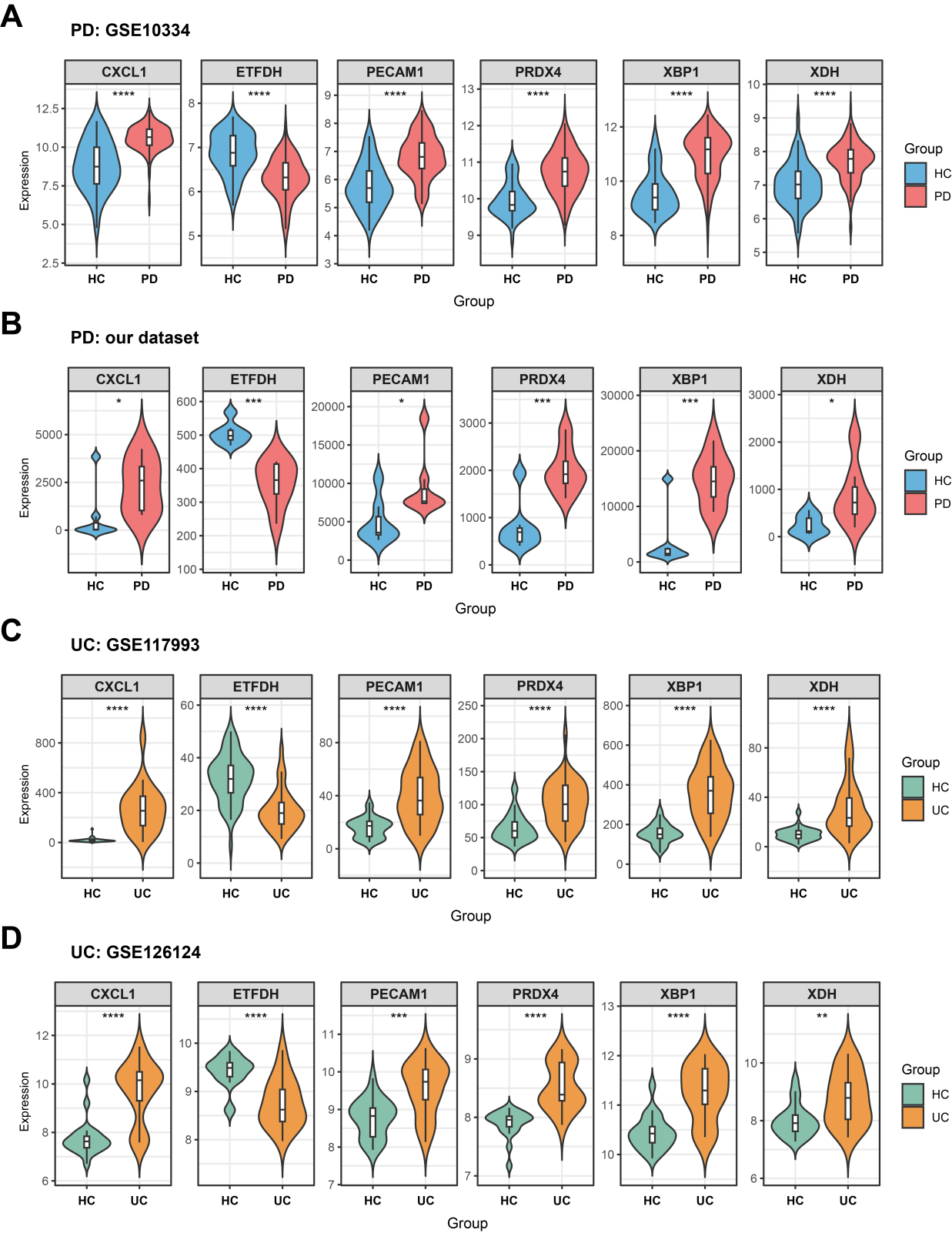
**

**Supplementary Figure 1. Oxidative stress-related co-expressed genes shared between PD and UC.**

1. B. Expression levels of six oxidative stress-related co-expressed genes (CXCL1, ETFDH, PECAM1, PRDX4, XBP1, and XDH) in PD datasets comparing HC and PD patients.

C-D. Expression patterns of oxidative stress-related co-expressed genes in UC datasets.

**
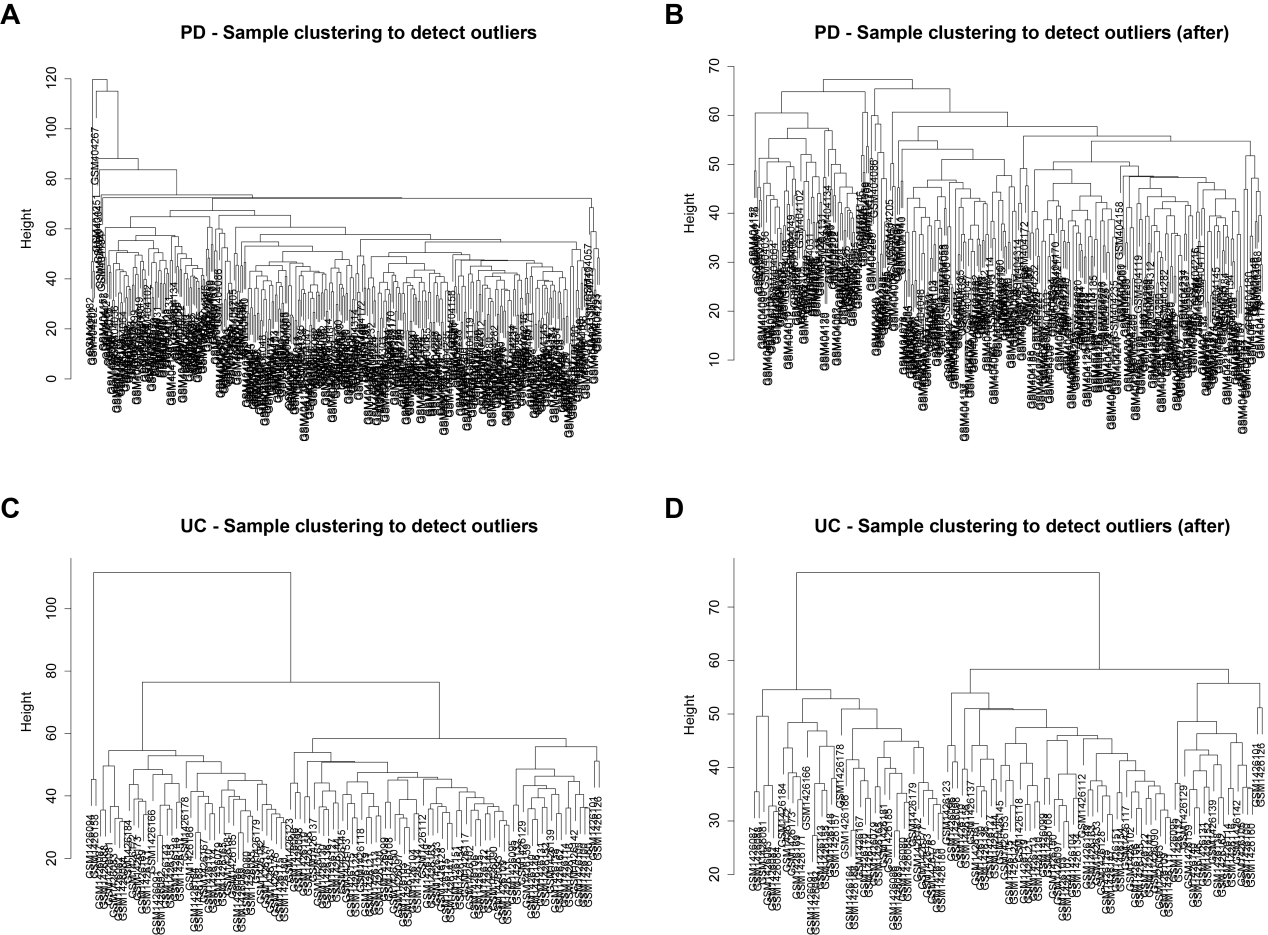
**

**Supplementary Figure 2. Sample clustering and outlier detection in PD and UC** **datasets.**

1. B. Hierarchical clustering dendrograms of the PD dataset pre- and post-outlier removal.

C-D. Hierarchical clustering dendrograms of the UC dataset pre- and post-outlier removal.

**
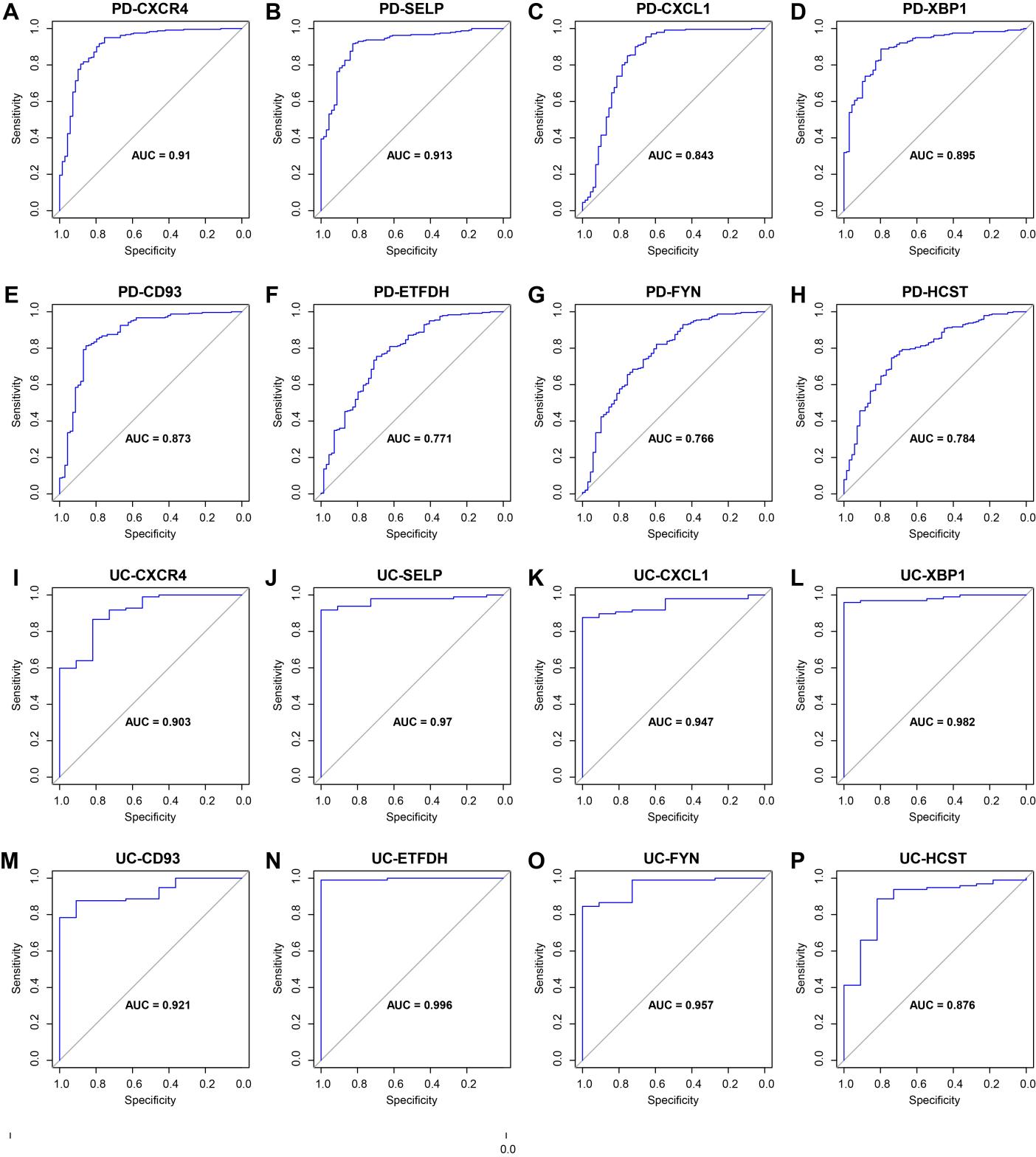
**

**Supplementary Figure 3. ROC curves of the shared hub genes.**

A-H. ROC curves of CXCR4, SELP, CXCL1, XBP1, CD93, ETFDH, FYN, and HCST in the PD dataset.

I-P. ROC curves of the shared hub genes in the UC dataset.

**
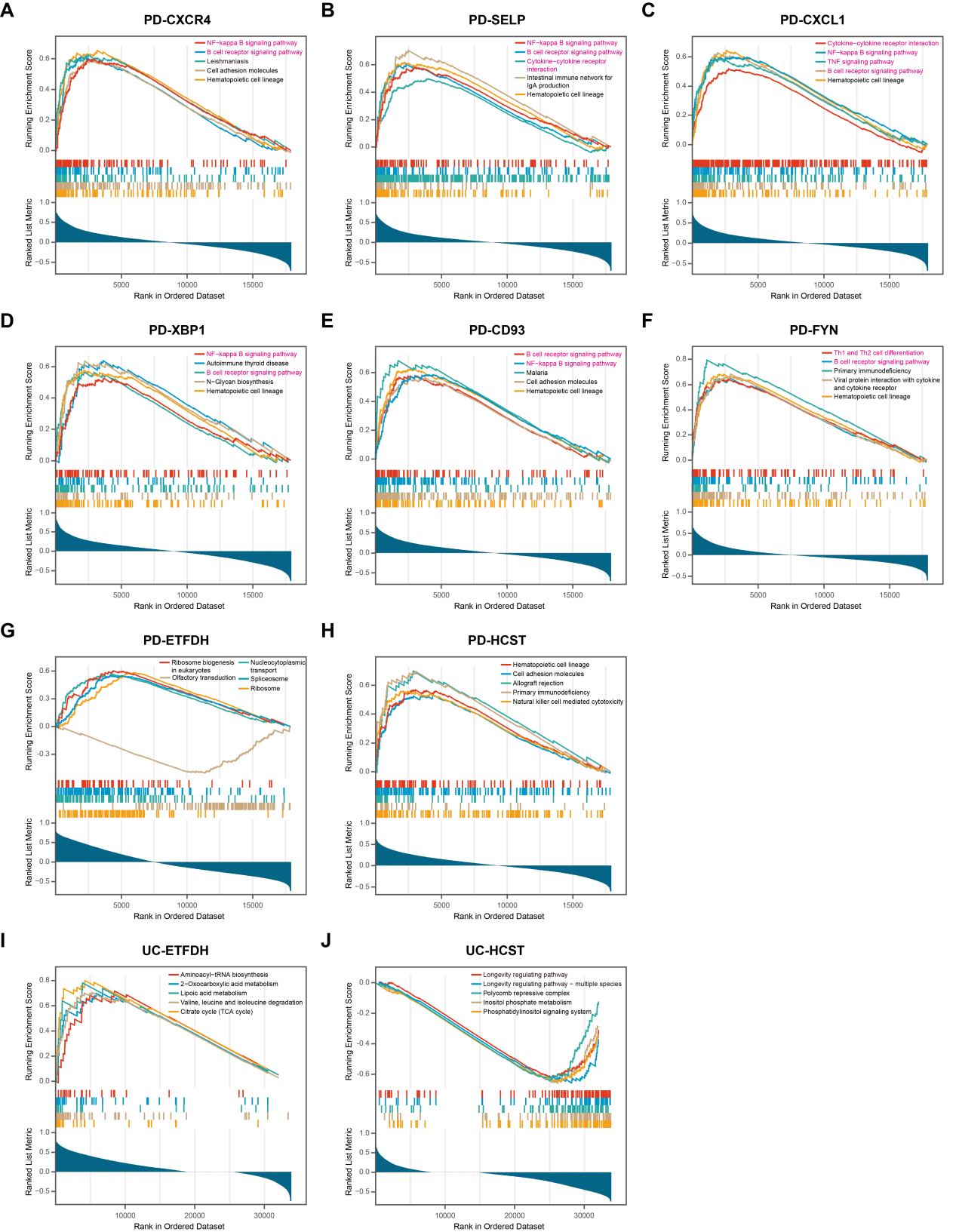
**

**Supplementary Figure 4. Gene Set Enrichment Analysis (GSEA) of hub genes in PD and UC.**

1. H. GSEA enrichment plots of hub genes in PD.
2. J. Enrichment plots of hub genes of in UC.

**
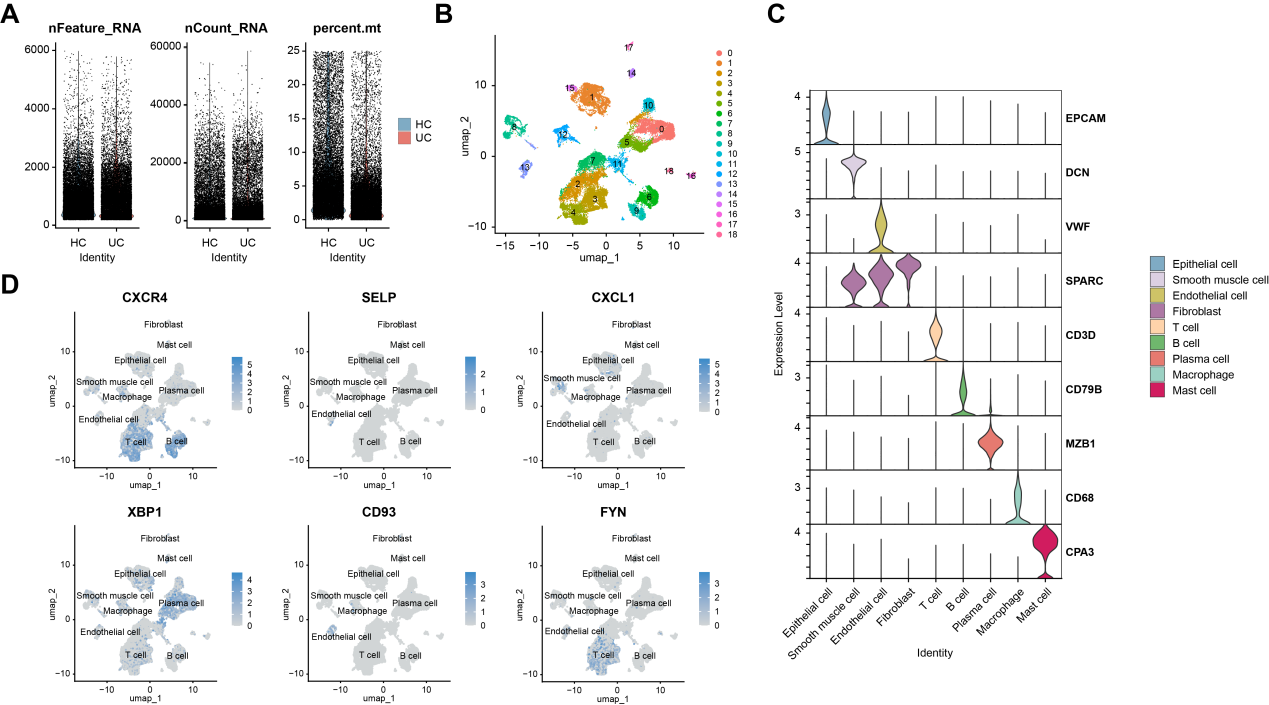
**

**Supplementary Figure 5. Validation of hub gene expression and cellular distribution in UC single-cell datasets.**

1. Distribution of nFeature_RNA, nCount_RNA, and percent.mt in intestinal samples from HC and UC patients.
2. UMAP visualization showing the classification of cells into 19 distinct clusters.
3. Violin plots of canonical marker genes for cell type annotation.
4. Expression patterns of hub genes across different cell types.

**
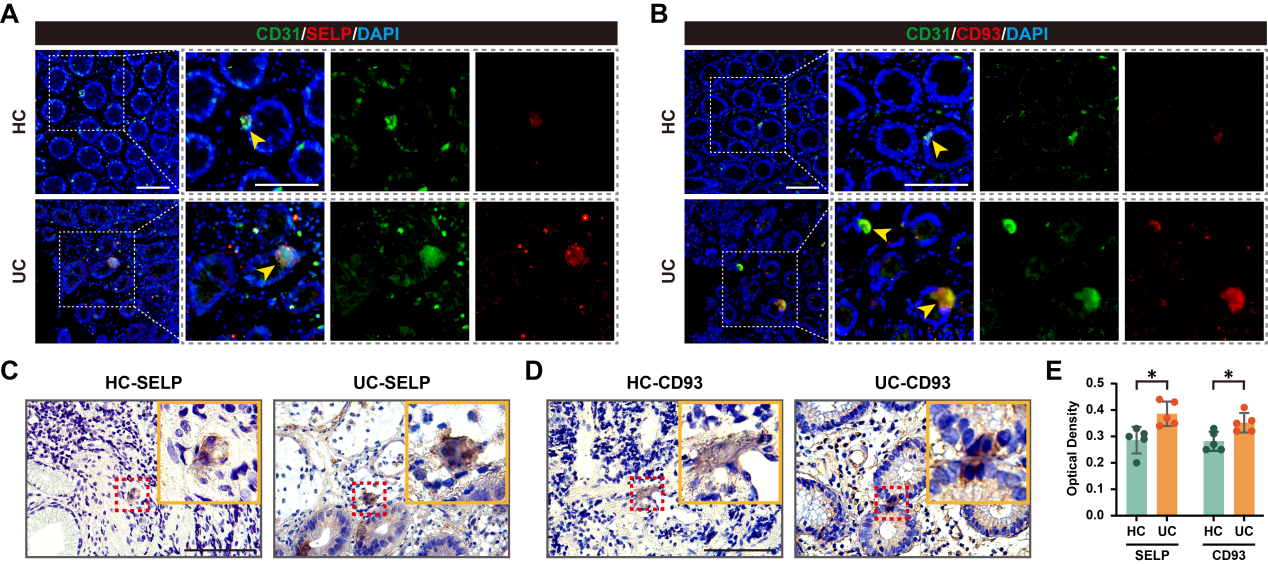
**

**Supplementary Figure 6. Validation of SELP and CD93 expression in UC clinical samples.**

A-B. IF staining showing the colocalization of SELP and CD93 with the endothelial marker CD31 in intestinal tissues from HC and UC patients. Nuclei were counterstained with DAPI; yellow arrows indicate regions of positive staining.

C-E. IHC staining and quantitative assessment of SELP and CD93 expression in HC and UC samples. Scale bar = 50 μm. Data are presented as mean ± SD (n = 5); *P < 0.05.
